# IL-24 promotes atopic dermatitis-like inflammation through driving MRSA-induced allergic responses

**DOI:** 10.1093/procel/pwae030

**Published:** 2024-05-16

**Authors:** Xinmin Qian, Meiyi Tong, Tianqing Zhang, Qingqing Li, Meng Hua, Nan Zhou, Wenwen Zeng

**Affiliations:** Institute for Immunology and School of Basic Medical Sciences, Tsinghua Medicine, Tsinghua University, Beijing 100084, China; Eight-year Medical Doctor Program, Chinese Academy of Medical Sciences & Peking Union Medical College, Beijing 100084, China; School of Life Sciences, Tsinghua University, Beijing 100084, China; School of Life Sciences, Tsinghua University, Beijing 100084, China; Institute for Immunology and School of Basic Medical Sciences, Tsinghua Medicine, Tsinghua University, Beijing 100084, China; Institute for Immunology and School of Basic Medical Sciences, Tsinghua Medicine, Tsinghua University, Beijing 100084, China; Institute for Immunology and School of Basic Medical Sciences, Tsinghua Medicine, Tsinghua University, Beijing 100084, China; Institute for Immunology and School of Basic Medical Sciences, Tsinghua Medicine, Tsinghua University, Beijing 100084, China; SXMU-Tsinghua Collaborative Innovation Center for Frontier Medicine, Shanxi Medical University, Taiyuan 030001, China; Tsinghua-Peking Center for Life Sciences, Beijing 100084, China; Beijing Key Laboratory for Immunological Research on Chronic Diseases, Beijing 100084, China

**Keywords:** IL-24, atopic dermatitis, MRSA, keratinocytes, allergic inflammation

## Abstract

Atopic dermatitis (AD) is a prevalent inflammatory skin disorder in which patients experience recurrent eczematous lesions and intense itching. The colonization of *Staphylococcus aureus* (*S*. *aureus*) is correlated with the severity of the disease, but its role in AD development remains elusive. Using single-cell RNA sequencing, we uncovered that keratinocytes activate a distinct immune response characterized by induction of *Il24* when exposed to methicillin-resistant *S. aureus* (MRSA). Further experiments using animal models showed that the administration of recombinant IL-24 protein worsened AD-like pathology. Genetic ablation of *Il24* or the receptor *Il20rb* in keratinocytes alleviated allergic inflammation and atopic march. Mechanistically, IL-24 acted through its heterodimeric receptors on keratinocytes and augmented the production of IL-33, which in turn aggravated type 2 immunity and AD-like skin conditions. Overall, these findings establish IL-24 as a critical factor for onset and progression of AD and a compelling therapeutic target.

## Introduction

Atopic dermatitis (AD) is a chronic and recurrent skin disease that is characterized by skin inflammation, barrier dysfunction, and intense itching (Langan et al., 2020). Globally, the prevalence of AD is estimated to be 15%–30% in children and 2%–10% in adults (Bieber, 2022; Weidinger et al., 2018). AD can have a significant impact on the life quality of patients, leading to disease-related disability and psychosocial consequences (Smith Begolka et al., 2021). Furthermore, AD is a known risk factor for the development of other allergic inflammatory diseases including asthma, food allergies, and allergic rhinitis (Hulpusch et al., 2021) and AD patients display a blood signature that indicates increased risk for cardiovascular disease (Brunner et al., 2017). While studies have demonstrated that the etiology and manifestations of AD result from complex interactions among genetic, immunological, and environmental factors (Stander, 2021), the precise multidimensional mechanism that underlies the pathogenesis of AD remains to be unearthed.

*S*. *aureus* is a commonly found Gram-positive bacterium that colonizes the skin of AD patients (Byrd et al., 2018; Lee et al., 2018; Ogonowska et al., 2020). Recent research has shown that *S*. *aureus* infection is strongly linked to AD pathology and contributes to skin inflammation (Byrd et al., 2017; Geoghegan et al., 2018; Leyva-Castillo et al., 2020; Liu et al., 2017; Meylan et al., 2017; Patrick et al., 2021; Simpson et al., 2018). The colonization rate is particularly high in patients with type 2 inflammatory features, such as high immunoglobulin E (IgE) and eosinophilia, and the degree of *S. aureus* colonization is correlated with worsened disease severity (Kong et al., 2012; Simpson et al., 2018; Tomczak et al., 2019). *S. aureus* is therefore proposed as a key factor of type 2 inflammation, notwithstanding the underlying causal relationship between the influence of *S. aureus* on the skin immune milieu and AD pathology remains to be elucidated.

The skin barrier plays a vital role in protecting our body against physical and infectious threats whilst adapting to changes in microbial colonization and invasion (Chen et al., 2018; Harris-Tryon and Grice, 2022). The integrity of the skin barrier depends on the concerted efforts of multiple cell types, including keratinocytes and immune cells, to maintain the skin structure and local immune homeostasis. However, aberrant barrier disruption, microbial infection, and inflammation can lead to disease conditions, such as AD, which is characterized by epidermal hyperplasia and elevated type 2 inflammatory cytokines like interleukin-4 (IL-4) (Carmi-Levy et al., 2011). Although the role of skin microbes in shaping barrier immunity is well established, host factors, particularly those that respond to *S*. *aureus* exposure, remain largely undefined. Investigating the skin-derived components that respond to microbial invasion and mediate immune reactions could provide valuable insights for developing targeted therapeutic interventions for AD.

In this study, we have uncovered interleukin-24 (IL-24), a cytokine expressed in keratinocytes and induced by MRSA infection, as a critical factor that drives the development of AD-like inflammation by dictating a type 2 immune responses. Single-cell RNA sequencing (scRNA-Seq) of the skin cells empowered us to delineate the skin immune reaction to MRSA invasion and identify IL-24 among the upregulated cytokines. We generated the conditional alleles of *Il24* and deleted them in keratinocytes by crossing them with *Krt14*^*Cre*^ mice (*Krt14*^*Cre*^; *Il24*^*fl*/*fl*^). By using the MC903-induced AD-like disease model, we observed a reduction of IL-4 and IgE in skin tissue and decreased IgE in serum in *Krt14*^*Cre*^; *Il24*^*fl*/*fl*^ mice compared to the wild-type group, in accordance with decreased disease severity, evidenced by ameliorated itching and improved skin barrier function. Consistently, genetic deletion of IL-24 receptor subunit *Il20rb* in keratinocytes showed similar phenotypes. Conversely, intradermal administration of recombinant IL-24 protein worked in the opposite manner. At the cellular level, we found that IL-24 primarily activated signal transducer and activator of transcription 3 (STAT3) in keratinocytes by acting on the heterodimeric receptors, leading to upregulation of interleukin-33 (IL-33) and the skewing of type 2 immune response. Our findings thus reveal a crucial role for skin IL-24 in promoting allergic inflammation and disease progression under AD-prone conditions.

## Results

### Skin HIMRSA application exacerbates AD-like inflammation and induces *Il24* expression

**Figure 1.**
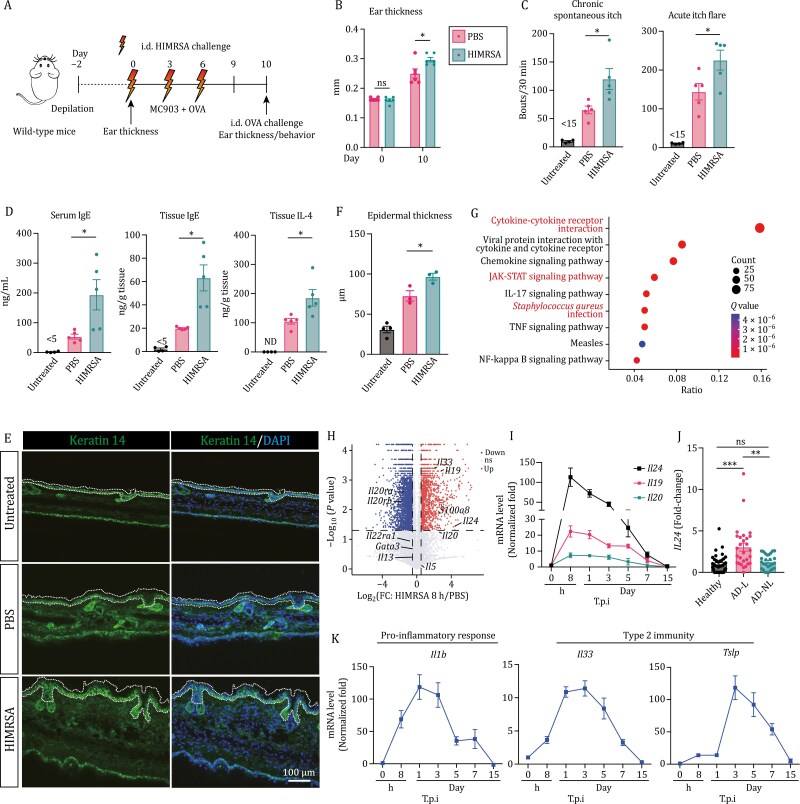
Skin HIMRSA application exacerbates AD-like inflammation and induces ***Il24*** expression. (A) Schematic of mouse AD-like disease model induction (MC903 + OVA) and experimental pipeline. Wild-type mice were intradermally (i.d.) challenged with PBS or HIMRSA every three days. (B) Ear thickness changes between Days 0 and 10 postmodel induction. Each data point represents the right ear thickness of one individual mouse. *n* = 5 mice. *P < 0.05 by unpaired *t*-test. ns, not significant. (C) Number of scratching bouts in HIMRSA-treated group and vehicle-treated control group before i.d. OVA challenge (chronic spontaneous itch; left) and following i.d. OVA challenge (acute itch flares; right) on Day 10 post model induction. Untreated group shows the basal level of scratching bouts. *n* = 4–5 mice, mean ± SEM. *P < 0.05 by unpaired *t*-test. (D) Serum IgE (left), tissue IgE (middle), and tissue IL-4 (right) in HIMRSA-treated group and vehicle-treated control group. Untreated group shows the basal level. *n* = 4–5 mice, mean ± SEM. *P < 0.05 by unpaired *t*-test. ND, not detected. (E and F) Representative immunofluorescent images showing the ear of HIMRSA-treated group and vehicle-treated control group. Antikeratin 14 antibody was used to label K14^+^ keratinocytes. Untreated group shows the basal level. The epidermal thickness was assessed and quantified in (F). *n* = 3–4 mice, mean ± SEM. *P < 0.05 by unpaired *t*-test. Dashed lines indicate the border of epidermis. (G) KEGG pathway analysis of DEGs in the skin from AD patients and healthy control in RNA-Seq datasets from the GEO database (GSE121212). (H) Volcano plot displaying the upregulated expression of IL-20 subfamily cytokines during HIMRSA application. Down, downregulation; ns, not significant; Up, upregulation. Dotted horizontal lines and dotted vertical lines represent, respectively, significance (FDR < 0.05) and fold change (log_2_ FC > |0.5|) thresholds. (I) Transcriptional analysis of the IL-20 subfamily cytokines in MRSA-treated skin of *wild-type* mice over the course of infection; results were presented by fold normalized to uninfected mice. *n* = 9–12 mice per time point, mean ± SEM. T.p.i., time post infection. (J) Expression of *IL24* in human skin samples analyzed from a publicly available dataset (GEO database, accession No. GSE121212). RNA-Seq analysis was performed in skin samples from 38 healthy controls and in lesional (AD-L) and non-lesional (AD-NL) skin samples from 27 patients with AD. Data were normalized to the gene *HPRT*. **P < 0.01, ***P < 0.001 by unpaired *t*-test. ns, not significant. (K) Transcriptional analysis of *Il1b*, *Il33,* and *Tslp* in MRSA-treated skin of wild-type mice over the course of infection; results were presented by fold normalized to uninfected mice. *n* = 9–12 mice per time point, mean ± SEM. T.p.i., time post infection.

Lesional skin from AD patients is often heavily colonized with *S*. *aureus*, showing the positive correlation between the bacterial burden and disease severity (Geoghegan et al., 2018; Higaki et al., 1999; Leyden et al., 1974; Leyva-Castillo et al., 2020; Tauber et al., 2016). To assess the effects of *S*. *aureus* colonization on AD, we first introduced heat-inactivated MRSA (HIMRSA) into an inducible mouse AD-like disease model which mimics the exposure to *S*. *aureus* invasion (Wang et al., 2021). Topical administration of MC903 and ovalbumin (OVA) resulted in the development of AD-like skin lesions, exhibiting skin redness and itching behavior (Video S1). When HIMRSA was applied through cutaneous administration (Fig. 1A), we observed that the disease conditions were accentuated. The mice displayed increased ear thickness (Fig. 1B), and intensified chronic and acute itch in the HIMRSA-treated group compared with vehicle-treated control group (Fig. 1C). Meanwhile, the levels of IgE increased in serum and lesional tissues were assessed by ELISA (Fig. 1D), indicative of heightened allergic reaction. In addition, IL-4 was elevated in lesional tissues upon HIMRSA treatment (Fig. 1D), consistent with an enhanced type 2 immune response. Histochemical analysis showed increased epidermal thickness (Fig. 1E and 1F). The results collectively validated that HIMRSA treatment could recapitulate the effects of *S*. *aureus* colonization on the deterioration of disease conditions.

To investigate the mechanism by which HIMRSA exacerbates disease conditions, we explored the transcriptional changes that occur during model induction. We performed RNA sequencing (RNA-Seq) analysis and profiled the genome-wide transcriptome at 8 and 24 h post-HIMRSA treatment, with a vehicle-treated group serving as control. Gene Ontology (GO) in biological process and Kyoto Encyclopedia of Genes and Genomes (KEGG) pathway enrichment analysis of overlapping differentially expressed genes (DEGs) revealed a pattern of *S*. *aureus* infection and the initiation of CD4^+^ T helper cell immune responses with the type 1 (Th1), type 2 (Th2) and type 17 (Th17) subtype signatures (Fig. S1A). Additionally, there was a significant enrichment of biological processes related to innate immune response, cell migration, phosphorylation, cell proliferation, and wound healing (Fig. S1B). Analysis of the RNA-Seq dataset [Gene Expression Omnibus (GEO) database: accession No. GSE121212] from AD patient-derived skin tissues also demonstrated a prevalence of *S*. *aureus* in the lesional skin and that the immune responses were pronounced, as evidenced by GO enrichment and KEGG pathway analysis (Figs. 1G and S1C).

The existence of a shared feature between *S. aureus* infection and associated immune response in both mice and patients motivated us to examine potential factors that might be induced by *S*. *aureus* infection and play critical roles in the pathogenesis of AD. Interestingly, global transcriptional profiling revealed that the expression of interleukin-20 subfamily cytokines, comprising *Il19* (*interleukin-19*), *Il20* (*interleukin-20*), and *Il24*, was increased during HIMRSA application (Figs. 1H and S1D). Among them, *Il24* presented the largest fold-change and highest transcription level with *Il19* being second and *Il20* being barely detectable.

The upregulation of IL-20 subfamily cytokines was confirmed by reverse transcription-quantitative PCR (RT-qPCR). Their transcriptional induction was detected within 8 h following MRSA application (Fig. 1I). Consistent with the observation in RNA-Seq analysis, the expression of the cytokines peaked at 8 h post-infection and gradually declined to basal level in the following 15 days. Notably, *Il24* exhibited the greatest and most persistent response to the infection among the family members (Fig. 1I). Further, by cutaneous application of live MRSA or HIMRSA, we found that *Il24* can be upregulated in a bacterial burden-dependent manner (Fig. S1E). In contrast, some other common pathogens and allergens, e.g., *Listeria monocytogenes*, house dust mite (HDM), and papain, were incapable of inducing *Il24* (Fig. S1F). The morphology and hematoxylin and eosin (H&E) staining of the MRSA-infected lesional sites assessed from Days 0 to 15 showed a change from host defense (inflammation, Day 1) to wound healing (Day 5) and advance to recovery state (Day 15) (Fig. S1G). Further, analysis of lesional and non-lesional skin samples from AD patients revealed that the expression of *IL24* was significantly elevated in the lesional region of AD patients, which was about 3-fold higher than in healthy controls and non-lesional region of the patients, indicating that the upregulation of *IL24* was restricted to lesional skin and IL-24 played a potentially conserved regulatory role in human and mouse (Fig. 1J).

Additionally, we characterized the transcriptional dynamics of genes involved in the pro-inflammatory response (*interleukin-1β*, *Il1b*; *C-X-C motif chemokine ligand 1*, *Cxcl1*; and *S100 calcium-binding protein a8*, *S100a8*), antimicrobial signaling (*β-defensin 4*, *Defb4*), skin barrier function (*periostin*, *Postn*; *serpin family b member 3a*, *Serpinb3a*), and type 2 immunity (*interleukin-33*, *Il33*; *thymic stromal lymphopoietin*, *Tslp*) (Figs. 1K and S1H). We observed the induction of these genes within 3 days of treatment, followed by their gradual decline to baseline levels at Day 15 postinfection. Host defense response-associated genes, including *Cxcl1* and *Il1b*, quickly reached their peak expression levels within 8–24 h (Figs. 1K and S1H). Whereas, the expression of *Defb4* and *S100a8* transcripts sustained at high levels until the wound healing stage at Day 5 (Fig. S1H). *Postn* showed a transient upregulation, while *Serpinb3a* exhibited a relatively delayed upregulation, remaining high until Day 7 (Fig. S1H). The expression of *Il33* and *Tslp* transcripts, two key components driving type 2 immunity, peaked at Day 3 and persisted to Day 7 (Fig. 1K).

Overall, the results have unveiled a series of distinct skin immune reactions in response to MRSA colonization and an enhanced type 2 immune feature. Those findings have led us to investigate the role of IL-24 in AD-like disease conditions.

### Keratinocytes produce IL-24 upon MRSA infection

**Figure 2.**
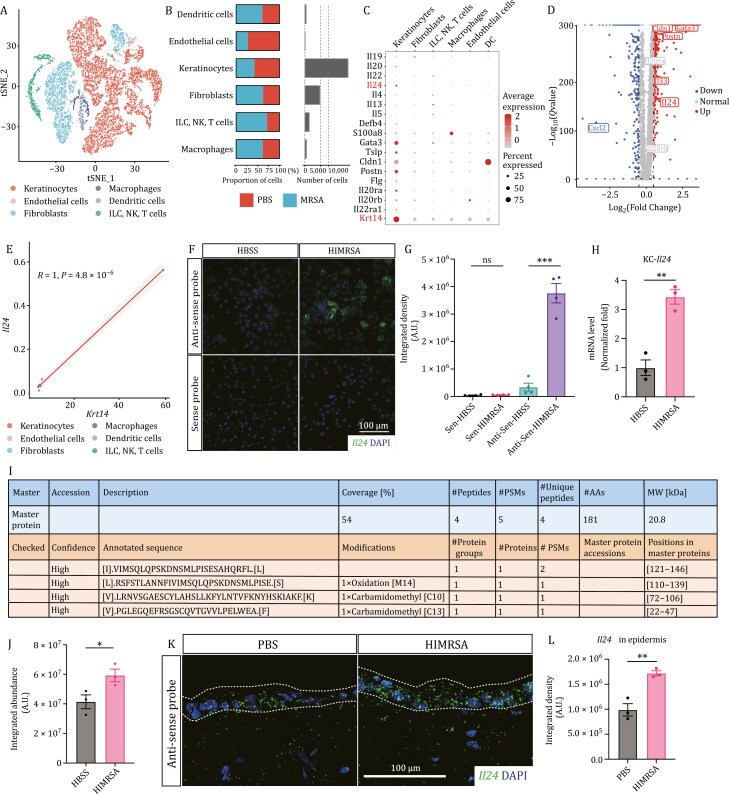
Keratinocytes are the main producer of IL-24 upon MRSA infection. (A) t-SNE plot displaying the 6 cell types from mouse skin. Cells were colored by types and annotated to the right. ILC, innate lymphoid cells; NK, natural killer cells. (B) Proportion and numbers of cells from the skin of mice with MRSA and vehicle application. (C) Dot plot showing feature genes related to infection and allergic responses for each cell type. (D) Volcano plot displaying genes that were differentially expressed in keratinocytes versus other cell types. Dotted horizontal lines and dotted vertical lines represent, respectively, significance (FDR < 0.05) and fold change (log_2_ FC > |0.5|) thresholds. (E) Correlation plots of mRNA levels of *Il24* and *Krt14* across different cell types. Scatterplots with estimated linear regression and 95% confidence interval; R, Pearson correlation. (F and G) *In situ* HCR images of primary keratinocytes at 12 h postindicated treatment, probed for *Il24* mRNA (antisense) and sense control. Integrated density of *Il24* mRNA labeled by green fluorescence was quantified in (G). *n* = 4, mean ± SEM. ***P < 0.001 by unpaired *t*-test. ns, not significant. (H) Expression of *Il24* in primary keratinocytes at 12 h postindicated treatment. Results were presented by fold normalized to control group. *n* = 3, mean ± SEM. **P < 0.01 by unpaired *t*-test. KC, keratinocyte. (I and J) Mass-spectrometry on specific IL-24 peptides from the supernatant of cultured primary keratinocytes at 24 h post indicated treatment. Integrated abundance of specific “IVIMSQLQPSKDNSMLPISESAHQRFLL” peptide was quantified in (J). *n* = 3, mean ± SEM. *P < 0.05 by unpaired *t*-test. MW, molecular weight; PSMs, peptide/spectrum matches; AAs, amino acids. (K and L) *In situ* HCR images of skin sections at 12 h post indicated treatment, probed for *Il24* mRNA (antisense). Integrated density of *Il24* mRNA labeled by green fluorescence was quantified in (L). *n* = 3, mean ± SEM. **P < 0.01 by unpaired *t*-test. Dashed lines indicate the border of epidermis.

The substantial increase in *Il24* upon MRSA treatment prompted us to investigate its associated cellular components and signaling events to understand its contribution to the course of AD-like inflammation. To accomplish this, we conducted single-cell RNA-sequencing (scRNA-Seq) analysis on skin cells collected from mice subjected to intradermal (i.d.) application of MRSA or vehicle control. By analyzing the expression matrix of their specific top markers, we identified a total of 17 cell clusters. The clusters were visualized through t-distributed stochastic neighbor embedding (t-SNE) (Fig. S2A and S2B). SingleR was implemented and validated to annotate cell identities. This allowed us to identify the corresponding clusters as epithelial cells (keratinocytes, 9 clusters), endothelial cells, fibroblasts (3 clusters), dendritic cells, macrophages (1 cluster, showing high scores for macrophages, monocytes, neutrophils, etc.), and innate lymphoid cells (ILC)\natural killer cells (NK)\T cells (2 clusters, showing high scores for ILC, NK, γδT, NKT, mast cells, eosinophils, basophils, and B cells) (Figs. 2A and S2C). Comparing the skin tissues infected with MRSA to the vehicle-treated ones, we found that there was an infiltration of immune cells in response to the infection (Figs. 2B and S2D). This resulted in an increase in the immune cell population such as dendritic cells, macrophages, and ILC/NK/T cells in the infected tissue (Figs. 2B and S2D). Consequently, the frequency of tissue-resident cells such as epithelial cells and endothelial cells decreased (Fig. 2B). Following the infection, there was an increase in fibroblasts that could aid in the generation of connective tissue (Fig. 2B). This, in turn, could provide a support system for cellular activity and facilitate prompt tissue repair (Davidson et al., 2021).

In agreement with the RNA-Seq data, *Il24* showed the highest expression among the IL-20 subfamily members and was primarily detected in keratinocytes (Fig. 2C). Additionally, two subunits of its receptor complex, *Il20ra* and *Il22ra1*, exhibited a unique expression pattern in keratinocytes and had a strong correlation with the gene expression of *Krt14* (*R* = 1, *P* = 4.1 × 10^−6^, and *R* = 0.96, *P* = 0.0027, respectively) (Fig. S2E and S2F). Conversely, *Il20rb*, the shared subunit, displayed a relatively ubiquitous expression pattern (*R* = 0.28, *P* = 0.59) (Fig. S2E and S2F). Our data-derived volcano plots across different cell types demonstrated that keratinocytes had a significant upregulation of *Il24* (Fig. 2D). Furthermore, the expression of *Il33* and *Tslp* highly correlated with keratinocytes and *Krt14* expression (*R* = 0.98, *P* = 6 × 10^−4^, and *R* = 0.91, *P* = 0.013, respectively) (Fig. S2F). The expression pattern of *Il24* remarkably overlapped with that of *Krt14* (Fig. 2E), strongly indicating that keratinocytes are the primary producers of IL-24 in response to MRSA exposure.

In parallel, we anatomically separated the skin tissues into layers post-HIMRSA application by peeling off the hypodermis and digesting the epidermis and dermis. This allowed us to characterize the enrichment of individual genes in specific tissue types. Pertinent to prior results, *Il24* showed the highest expression level in the epidermis, exhibiting a similar pattern to that of *Krt14* (Fig. S2G and S2H). Moreover, we cultured primary murine keratinocytes and observed HIMRSA-induced upregulation of *Il24*, which was confirmed by *in situ* hybridization chain reaction (HCR) assay and RT-qPCR (Fig. 2F–H). By performing mass spectrometry experimentation, we captured IL-24 protein fragments in the supernatant of HIMRSA-stimulated keratinocytes (Fig. 2I), which was quantified in Fig. 2J. Additionally, by leveraging HCR, we verified the expression pattern of *Il24* in the skin during HIMRSA application (Fig. 2K and 2L). Taken together, these findings strongly indicate that keratinocytes are the primary producers of IL-24 upon MRSA infection.

### Keratinocyte-derived IL-24 drives AD-like inflammation and promotes asthma-like symptoms

To determine the role of IL-24 in influencing disease conditions, we generated a conditional allele of *Il24* (*Il24*^*fl*/*fl*^) and crossed it to *Krt14*^*Cre*^ mice (*Krt14*^*Cre*^; *Il24*^*fl*/*fl*)^ to ablate *Il24* in keratinocytes (Strategy shown in Fig. S3A). The littermate *Il24*^*fl*/*fl*^ mice served as control in the following characterization. Keratinocyte-specific deletion of *Il24* was verified by qPCR on transcript and mass-spectrometry on protein post MRSA infection (Fig. S3B and S3C). The deletion did not alter the morphology of back and facial skin and cast no impact on the proliferation and differentiation of keratinocytes at steady states. The epidermal thickness was also comparable between the two groups (Fig. S3D–F).

**Figure 3.**
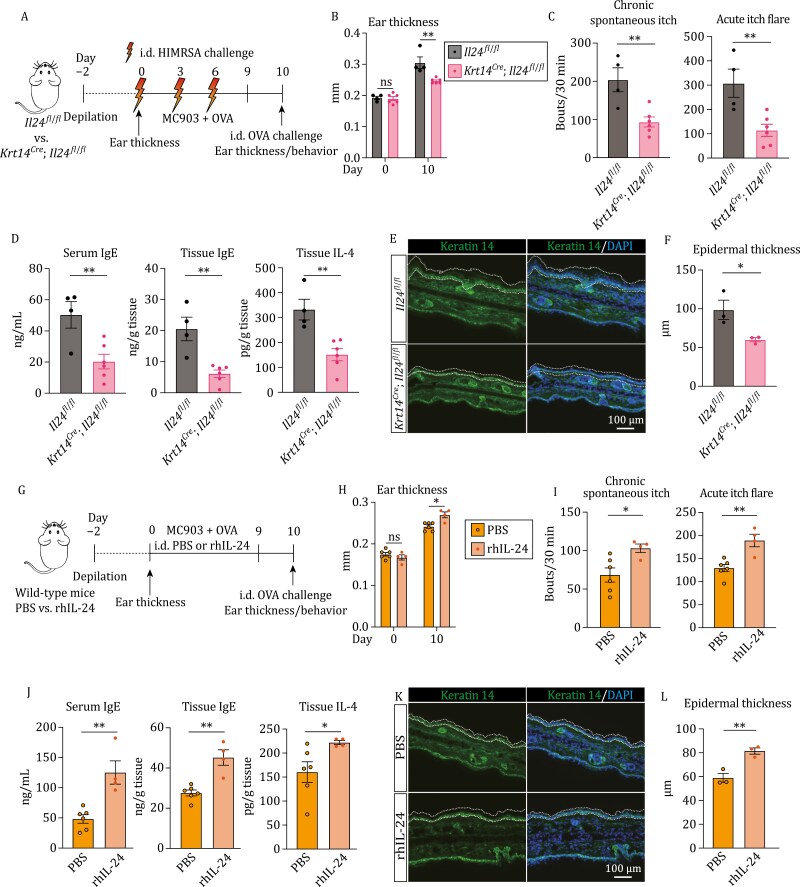
Keratinocyte-derived IL-24 drives AD-like inflammation. (A) Schematic of mouse AD-like disease model (MC903 + OVA) and experimental pipeline. *Il24f*^*fl*/*fl*^ and *Krt14*^*Cre*^; *Il24f*^*fl*/*fl*^ mice were intradermally (i.d.) challenged with HIMRSA every three days. (B) Ear thickness changes between Days 0 and 10 postmodel induction. Each data point represents the right ear thickness of one individual mouse. *n* = 4–6 mice. **P < 0.01 by unpaired *t*-test. ns, not significant. (C) Number of scratching bouts in *Il24f*^*fl*/*fl*^ and *Krt14*^*Cre*^; *Il24f*^*fl*/*fl*^ mice before i.d. OVA challenge (chronic spontaneous itch; left) and following i.d. OVA challenge (acute itch flares; right) on Day 10 post model induction. *n* = 4–6 mice, mean ± SEM. **P < 0.01 by unpaired *t*-test. (D) Serum IgE (left), tissue IgE (middle), and tissue IL-4 (bottom) in *Il24f*^*fl*/*fl*^ and *Krt14*^*Cre*^; *Il24f*^*fl*/*fl*^ mice. *n* = 4–6 mice, mean ± SEM. **P < 0.01 by unpaired *t*-test. (E and F) Representative immunofluorescent images showing the ear of *Il24f*^*fl*/*fl*^ and *Krt14*^*Cre*^; *Il24f*^*fl*/*fl*^ mice on Day 10 post model induction. Antikeratin 14 antibody was used to label K14^+^ keratinocytes. The epidermal thickness was assessed and quantified in (F). *n* = 3 mice, mean ± SEM. *P < 0.05 by unpaired *t*-test. Dashed lines indicate the border of epidermis. (G) Schematic of mouse AD-like disease model (MC903 + OVA) and experimental pipeline. Wild-type mice were intradermally (i.d.) treated with PBS or rhIL-24 every day. (H) Ear thickness change between Days 0 and 10 post-model induction. Each data point represents the right ear thickness of one individual mouse. *n* = 4–6 mice. *P < 0.05 by unpaired *t*-test. ns, not significant. (I) Number of scratching bouts in rhIL-24-treated group and vehicle-treated control group before i.d. OVA challenge (chronic spontaneous itch; left) and following i.d. OVA challenge (acute itch flares; right) on Day 10 post model induction. *n* = 4–6 mice, mean ± SEM. *P < 0.05, **P < 0.01 by unpaired *t*-test. (J) Serum IgE (left), tissue IgE (middle), and tissue IL-4 (right) in rhIL-24-treated group and vehicle-treated control group. *n* = 4–6 mice, mean ± SEM. *P < 0.05, **P < 0.01 by unpaired *t*-test. (K and L) Representative immunofluorescent images showing the ear of rhIL-24-treated group and vehicle-treated control group. Antikeratin 14 antibody was used to label K14^+^ keratinocytes. The epidermal thickness was assessed and quantified in (L). *n* = 3 mice, mean ± SEM. **P < 0.01 by unpaired *t*-test. Dashed lines indicate the border of epidermis.

When introducing the MC903-induced AD model combined with HIMRSA treatment (Fig. 3A), we found that abrogation of *Il24* alleviated the disease conditions, marked by reduced ear thickness in *Krt14*^*Cre*^; *Il24*^*fl*/*fl*^ group (Fig. 3B). Both chronic spontaneous itch and acute itch flare were mitigated markedly (Fig. 3C). Consistently, the levels of serum and tissue IgE were reduced, and the associated allergic inflammation was dampened, showing lower level of tissue IL-4 in *Krt14*^*Cre*^; *Il24*^*fl*/*fl*^ group than control group (Fig. 3D). Moreover, decreased epidermal thickness was detected by immunofluorescent staining (Fig. 3E and 3F).

Next, we interrogated whether alleviated AD-like inflammation caused by *Il24* depletion could further the remission of atopic march, e.g., asthma. We intranasally treated mice with OVA to induce asthma-like symptoms after epicutaneous sensitization (Fig. S4A). OVA-specific IgE in serum and lung were reduced in *Krt14*^*Cre*^; *Il24*^*fl*/*fl*^ group (Fig. S4B and S4C). And the allergic inflammation was diminished, presenting a lower level of IL-4 in the lung (Fig. S4D). Whole-body plethysmograph (WBP) analysis of Penh (Enhanced pause) was used to evaluate airway constriction (Hamelmann et al., 1997). A decreased level of Penh after OVA induction was observed in *Krt14*^*Cre*^; *Il24*^*fl*/*fl*^ group, indicative of better lung function (Fig. S4E). In addition, pulmonary allergic inflammation was assessed by immunofluorescent staining with anti-SiglecF antibody and *Krt14*^*Cre*^; *Il24*^*fl*/*fl*^ group showed a reduced signal intensity (Fig. S4F and S4G).

Collectively, the data suggest that IL-24 derived from keratinocytes drives the type 2 immune response and aggravates AD and asthma-like conditions.

### Cutaneous administration of recombinant IL-24 exacerbates AD-like inflammation

To further validate the *in vivo* effect on AD-like inflammation induced by IL-24, we administrated recombinant human IL-24 (rhIL-24) protein to MC903-induced AD model through daily intradermal injection, with vehicle-injected group as control (Fig. 3G). Consistently, the recombinant IL-24 protein aggravated the pathological conditions, showing thickened ear (Fig. 3H) and intensified itch (Fig. 3I). Further, the levels of serum and lesional tissue IgE were higher in rhIL24-treated group (Fig. 3J). And the allergic inflammation was augmented, presenting higher level of IL-4 in tissues (Fig. 3J). In addition, immunofluorescent staining showed that epidermal thickness was increased (Fig. 3K and 3L).

Moreover, we applied rhIL-24 protein to the skin of *Krt14*^*Cre*^; *Il24*^*fl*/*fl*^ mice to determine whether the recombinant protein could functionally reverse the alleviated disease conditions (Fig. S5A). The group with rhIL-24 treatment showed even thicker ear and slightly more severe acute itch flare than the control, while chronic spontaneous itch was relatively comparable (Fig. S5B and S5C). Serum IgE levels were moderately increased, and decreased tissue IgE and IL-4 levels observed in knockout mice were restored (Fig. S5D). Immunofluorescent staining confirmed enhanced epidermal thickness (Fig. S5E and S5F). These data provide further evidence that IL-24 produced by keratinocytes during MRSA infection deteriorates disease conditions.

### IL-24 activates JAK-STAT pathway in keratinocytes in AD model

**Figure 4.**
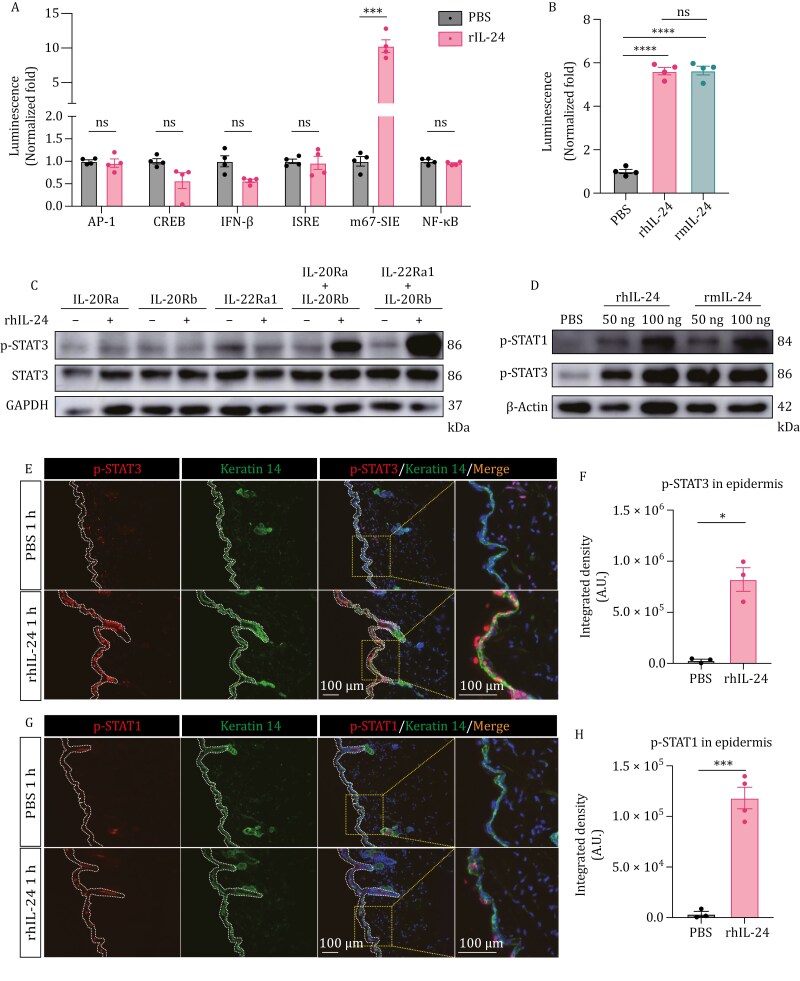
IL-24 activates JAK-STAT pathway in keratinocytes. (A) Characterization of different signaling pathways determined by luciferase reporter assay in HEK293T cells co-expressing the receptors. AP-1, activator protein-1; CREB, cyclic-AMP response binding protein; IFN-β, interferon-β; ISRE, interferon-stimulated response elements; m67-SIE, m67-sis inducible element; NF-κB, nuclear factor-κB. *n* = 4, mean ± SEM. ***P < 0.001 by unpaired *t*-test. ns, not significant. (B) Luciferase reporter assay of m67-SIE responding to rhIL-24 or rmIL-24 stimulation in HEK293T cells co-expressing the receptors. Relative luciferase activities driven by m67-SIE were measured at 12 h post rIL-24 treatment. *n* = 4, mean ± SEM. ****P < 0.0001 by unpaired *t*-test. ns, not significant. (C) Immunoblot analysis of HEK293T cells expressing indicated receptors responding to rhIL-24 stimulation. (D) Immunoblot analysis of skin samples from wild-type mice treated with indicated rIL-24 or vehicle-control. (E–H) Representative immunofluorescent images showing the skin of rhIL-24-treated group and vehicle-treated control group for 1 h. Zoomed views on the right indicate the regions outlined by orange dashed rectangles. Quantification was shown in (F and H) indicating the phosphorylation of STAT3 and STAT1, respectively. Antikeratin 14 antibody was used to label K14^+^ keratinocytes. *n* = 3–4 mice, mean ± SEM. *P < 0.05, ***P < 0.001 by unpaired *t*-test. Dashed lines indicate the border of epidermis.

Next, we set to determine the signaling events governing the aggravation of the disease conditions by IL-24. Previous studies suggest that IL-24 can activate Janus-activated tyrosine kinase (JAK)-STAT1/3 in human keratinocytes (Dumoutier et al., 2001; Wang et al., 2002). Firstly, we confirmed that in the presence of the receptor complex, IL-24 can activate the STAT1/3 signaling cascade, as validated through a luciferase assay conducted in HEK293T cells. Various reporters, such as activator protein-1 (AP-1), cyclic-AMP response element binding protein (CREB), interferon-β (IFN-β), interferon-stimulated response elements (ISRE), m67-sis inducible element (m67-SIE), and nuclear factor-κB (NF-κB), were delivered to verify whether a series of immune responses were activated in the process. Upon addition of rhIL-24 to cultured cells, a significant increase in m67-SIE reporter activity was detected, while no noticeable activation of other reporters was found (Fig. 4A). Both human and mouse recombinant IL-24 could trigger the STAT1/3 signaling, indicating conservation of the signaling function in both species (Fig. 4B). Moreover, a heterodimeric receptor composed of IL-20Ra/IL-20Rb or IL-22Ra1/IL-20Rb was essential for the IL-24 response, and the lack of either subunit was insufficient to trigger STAT3 phosphorylation (Fig. 4C). *In vivo*, we detected p-STAT3 in mouse skin samples subjected to either HIMRSA or MRSA treatment (Fig. S6A and S6B). However, p-STAT1 was barely detectable (data not shown), indicating a predominant STAT3 activation in keratinocytes, in response to IL-24.

Consistent with the response to IL-24 in keratinocytes, scRNA-Seq data showed that the expression of *Il20ra* and *Il22ra1* was highly correlated with *Krt14* marking keratinocytes (Figs. 2C and S2F). Additionally, our analysis of primary keratinocytes showed a decrease in receptor expression after plating by Day 2 (Fig. S6C), which aligns with the findings of Liu et al. demonstrating that IL-22Ra1\IL-20Rb receptors are largely silenced *in vitro* (Liu et al., 2023). Therefore, we aimed to investigate the signaling events induced by rhIL-24 in keratinocytes immediately postseeding. A drastically increased signal of p-STAT3 and a slight increase of p-STAT1 were detected (Fig. S6D and S6E). Notably, the receptors were expressed at very low levels and almost undetectable determined by scRNA-Seq in immune cell subpopulations (Fig. 2C). As a result, when rhIL-24 was added to the immune cells such as SiglecF^+^ eosinophils, SiglecF^-^ immune cells, CD11c^+^, CD3^+^, CD11b^+^ cells isolated from splenocytes and lymph nodes (LN), or bone marrow-derived dendritic cells (BMDC), p-STAT3 did not show a significant change assessed by immunoblot (Fig. S6F–K). When the naïve CD4 T cells were isolated and activated by anti-CD3 and anti-CD28 antibodies *in vitro*, no significant change in p-STAT3 was observed with the administration of rhIL-24 (Fig. S6L). In addition, when the cells were activated and induced to Th2 cell differentiate, rhIL-24 did not alter the expression of GATA binding protein 3 (GATA3), indicating rhIL-24 did not significantly impact the differentiation of CD4 T cells to Th2 cell subtype directly (Fig. S6M). The data together indicate that IL-24 predominantly signals keratinocytes to engage the STAT3 signaling pathway.

**Figure 5.**
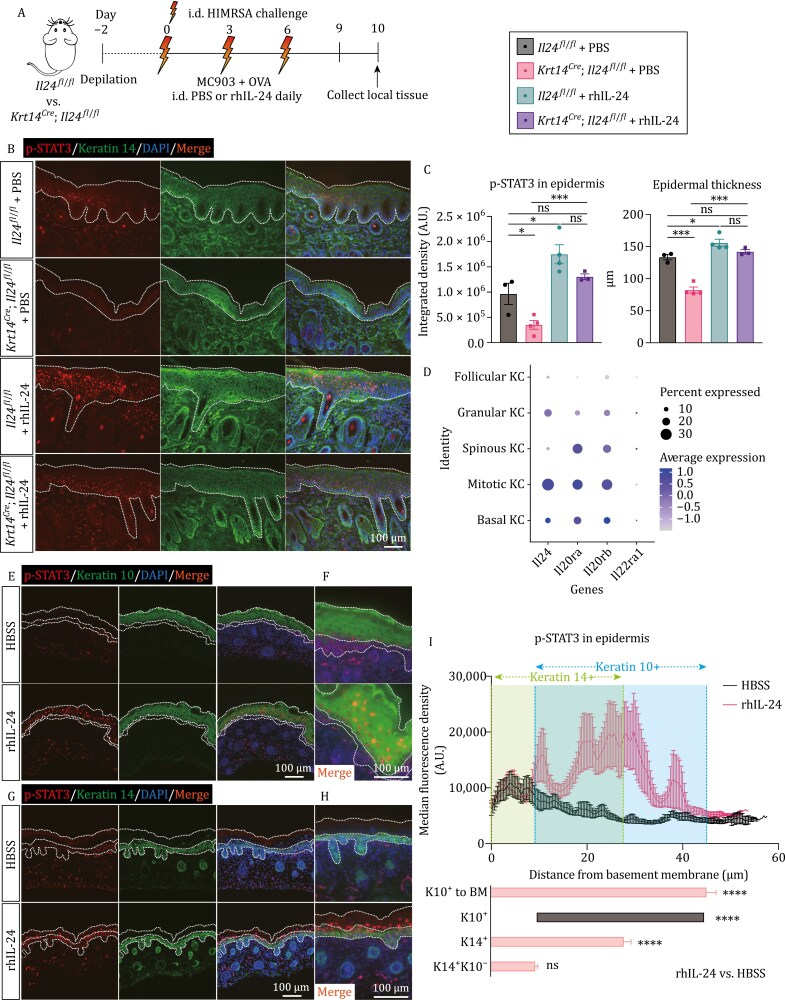
IL-24 exacerbates AD-like inflammation by activating JAK-STAT pathway primarily in K10^+^ keratinocytes. (A) Schematic of mouse AD-like disease model (MC903 + OVA) and experimental pipeline. *Il24f*^*fl*/*fl*^ and *Krt14*^*Cre*^; *Il24f*^*fl*/*fl*^ mice were intradermally (i.d.) treated respectively with PBS or rhIL-24 every day. (B and C) Representative immunofluorescent images showing the p-STAT3 signals from *Il24f*^*fl*/*fl*^ and *Krt14*^*Cre*^; *Il24f*^*fl*/*fl*^ mice with indicated treatment postmodel induction. Quantification of p-STAT3 and epidermal thickness were shown in (C). Antikeratin 14 antibody was used to label K14^+^ keratinocytes. *n* = 3–4 mice, mean ± SEM. *P < 0.05, ***P < 0.001 by unpaired *t*-test. ns, not significant. Dashed lines indicate the border of epidermis. (D) Dot plot showing the expression of *Il24* and receptor genes for keratinocyte-subclusters. KC, keratinocyte. (E and F) Representative immunofluorescent images showing the p-STAT3 signals of the skin from postnatal Day 0 (p0) mouse with indicated treatment for 1 h. Zoomed views were shown in (F). Antikeratin 10 antibody was used to label K10^+^ keratinocytes. Dashed lines indicate the border of epidermis and K10^+^ keratinocytes. (G and H) Representative immunofluorescent images showing the p-STAT3 signals of the skin from p0 mouse with indicated treatment for 1 h. Zoomed views were shown in (H). Antikeratin 14 antibody was used to label K14^+^ keratinocytes. Dashed lines indicate the border of epidermis and K14^+^ keratinocytes. (I) Quantification of p-STAT3 signals in epidermis (upper) across different types of keratinocytes (bottom) after rhIL-24 treatment for 1 h. *n* = 10, mean ± SEM. ****P < 0.0001 by unpaired *t*-test. ns, not significant. BM, basement membrane.

Next, we interrogated the IL-24-STAT signaling in the AD mouse model. When applying rIL-24 to the skin of *wild-type* mice through intradermal administration, we detected strong p-STAT3 and weak p-STAT1 signals in the epidermis (Fig. 4D–H), both of which were in a dose-dependent manner. Conspicuously, in the lesional skin tissues of AD mice, strong p-STAT3 and weak p-STAT1 signals were observed (Figs. 5B and S7A). Ablation of *Il24* decreased both signals, whereas rhIL-24 application showed the opposite effect (Figs. 5B, 5C, S7A and S7B). Meanwhile, the lesional epidermal thickness was consistent with the extent of STAT activation (Figs. 5C and S7B). Together, these data indicate that IL-24 triggers STAT activation in keratinocytes upon model induction, which is in line with disease severity.

### IL-24 functions through receptor complex in a paracrine way

The differential phosphorylation of STAT3 across different epidermal layers inspired us to determine whether keratinocytes-derived IL-24 functions in the autocrine/paracrine manner in the skin. Classifying the *Krt14*-marked keratinocytes into different subclusters in scRNA-Seq (Fig. S7C), we revealed that the expression of *Il24* and the receptor genes showed slightly different patterns. *Il24* was mostly expressed in mitotic cells from the basal layer, while the receptors, especially *Il22ra1*, were highly expressed in differentiated keratinocytes from spinous and granular layers marked by high levels of *Krt1* and *Krt10*, in addition to mitotic cells (Figs. 5D and S7C). Notably, IL-22Ra1 paired with IL-20Rb was the dominant receptor complex for signaling in the murine system (Kolumam et al., 2017). When the skin from Day 0 postnatal (p0) mice, which have easily distinguished layers (Li et al., 2017), was treated with rhIL-24 for 1 h, immunofluorescent staining showed that the increased p-STAT3 was predominantly located in keratin 10^+^ (K10^+^) keratinocytes, whereas K14^+^K10^−^ keratinocytes, which include the mitotic cells from basal layers, had slight but not significant increase (Fig. 5E–I; Videos S2 and S3). The results suggest that the differentiated keratinocytes in spinous and granular layers represent the major responsive populations to IL-24 produced by mitotic cells. Consistently, p-STAT1, though weaker, showed a similar pattern to p-STAT3 (Fig. S7D–H). These data implied that IL-24 produced by mitotic keratinocytes function in a paracrine way during MRSA infection.

**Figure 6.**
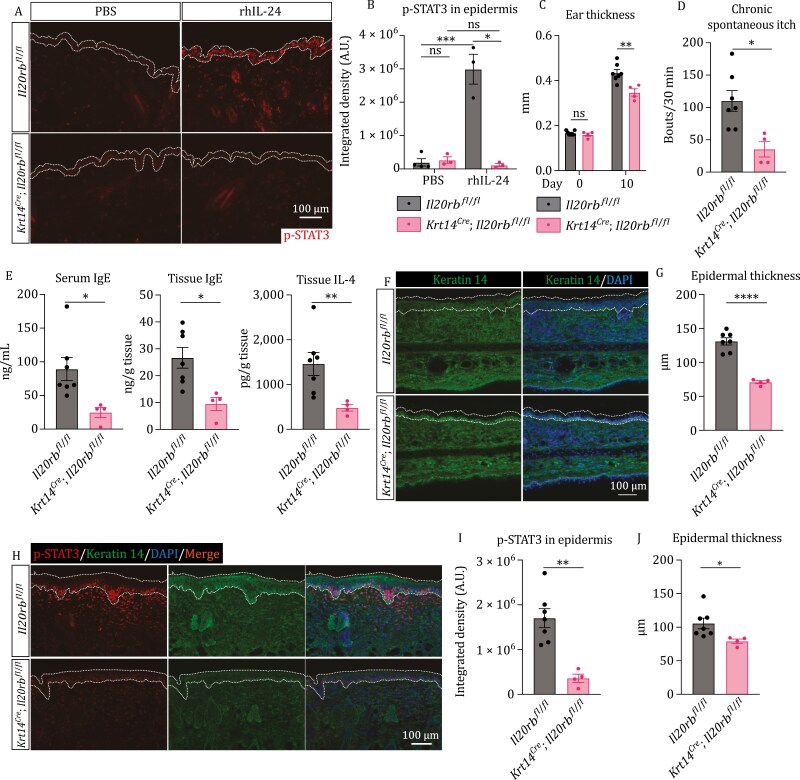
Keratinocyte-specific ***Il20rb*** depletion abrogates IL-24-STAT3 signaling and alleviates AD-like disease conditions. (A and B) Representative immunofluorescent images showing the p-STAT3 signals from *Il20rbf*^*fl*/*fl*^ and *Krt14*^*Cre*^; *Il20rbf*^*fl*/*fl*^ mice with indicated treatment for 1 h. Quantification of p-STAT3 in the epidermis was shown in (B). *n* = 3–4 mice, mean ± SEM. *P < 0.05, ***P < 0.001 by unpaired *t*-test. ns, not significant. Dashed lines indicate the border of epidermis. (C) Ear thickness change between Days 0 and 10 post model induction. Each data point represents the right ear thickness of one individual mouse. *n* = 4–7 mice. **P < 0.01 by unpaired *t*-test. ns, not significant. (D) Number of scratching bouts in *Il20rbf*^*fl*/*fl*^ and *Krt14*^*Cre*^; *Il20rbf*^*fl*/*fl*^ mice on Day 10 postmodel induction. *n* = 4–7 mice, mean ± SEM. *P < 0.05 by unpaired *t*-test. (E) Serum IgE (left), tissue IgE (middle), and tissue IL-4 (right) in *Il20rbf*^*fl*/*fl*^ and *Krt14*^*Cre*^; *Il20rbf*^*fl*/*fl*^ mice. *n* = 4–7 mice, mean ± SEM. *P < 0.05, **P < 0.01 by unpaired *t*-test. (F and G) Representative immunofluorescent images showing the ear of *Il20rbf*^*fl*/*fl*^ and *Krt14*^*Cre*^; *Il20rbf*^*fl*/*fl*^ mice on Day 10 post model induction. Antikeratin 14 antibody was used to label K14^+^ keratinocytes. The epidermal thickness was assessed and quantified in (I). *n* = 4–7 mice, mean ± SEM. ****P < 0.0001 by unpaired *t*-test. Dashed lines indicate the border of the epidermis. (H–J) Representative immunofluorescent images showing the p-STAT3 signals from *Il20rbf*^*fl*/*fl*^ and *Krt14*^*Cre*^; *Il20rbf*^*fl*/*fl*^ mice postmodel induction. Quantification of p-STAT3 and epidermal thickness were shown in (I and J), respectively. Antikeratin 14 antibody was used to label K14^+^ keratinocytes. *n* = 4–7 mice, mean ± SEM. *P < 0.05, **P < 0.01 by unpaired *t*-test. Dashed lines indicate the border of epidermis.

To provide further evidence for the important role of IL-24-receptors-STAT signaling in AD-like inflammation, we generated the conditional alleles of *Il20rb* (*Il20rb*^*fl*/*fl*^) and abrogated *Il20rb* in keratinocytes by crossing *Krt14*^*Cre*^; *Il20rb*^*fl*/*fl*^ (Fig. S8A). The specific deletion of *Il20rb* was verified by qPCR on genomic DNA and transcript levels (Fig. S8B and S8C). Upon rhIL-24 treatment, the activation of STAT3 was abolished (Figs. 6A, 6B, S8D and S8E). When the animals were subjected to the AD model as above (Fig. 3C), we found that *Il20rb* deletion mitigated the disease conditions, evidenced by reduced ear thickness and spontaneous itch in *Krt14*^*Cre*^; *Il20rb*^*fl*/*fl*^ group (Fig. 6C and 6D). Consistently, serum IgE, tissue IgE, and tissue IL-4 were reduced (Fig. 6E). Moreover, decreased epidermal thickness and STAT3 activation were observed (Fig. 6F–J).

In general, IL-24 activates JAK-STAT signaling pathway through its receptor complexes mainly in differentiated keratinocytes, of which IL-20Rb was necessary while IL-22Ra1/IL-20Rb was predominant, thus promoting allergic inflammation and disease conditions.

### IL-24 enhances IL-33 production and drives type 2 immunity

**Figure 7.**
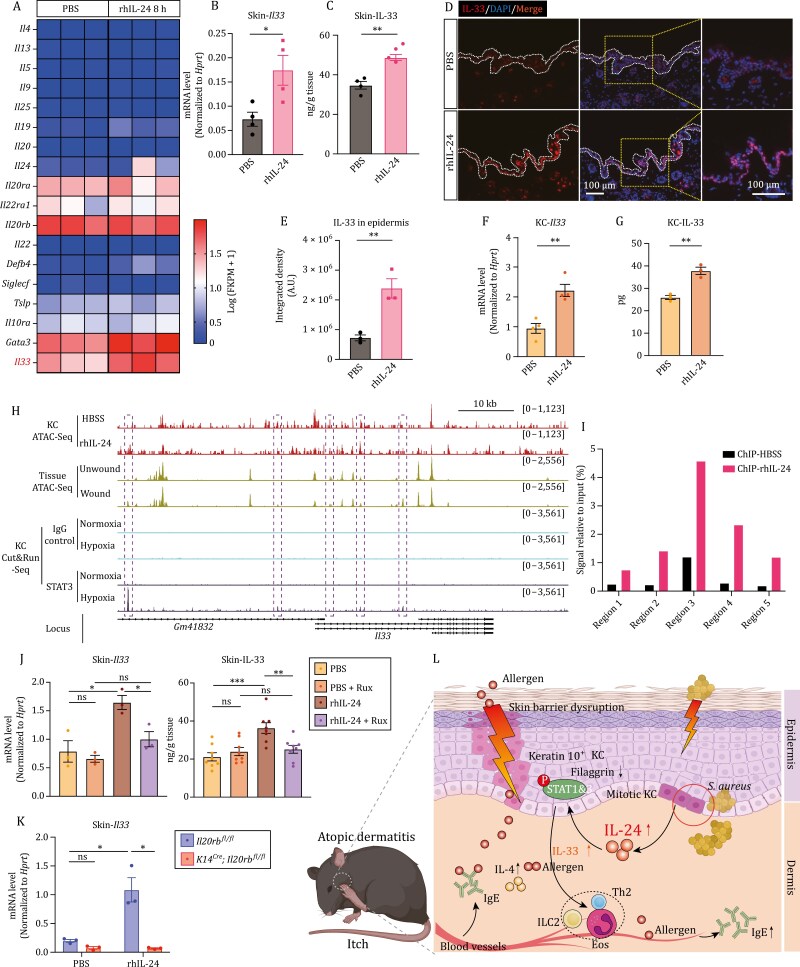
IL-24 enhances IL-33 production and drives type 2 immunity. (A) Heatmap showing the feature genes related to allergic responses in skin samples from rhIL-24-treated mice (8 h) and vehicle-control mice by RNA-Seq analysis. (B) Expression of *Il33* in the skin of wild-type mice with indicated treatment for 12 h. Relative expression level was normalized to *Hprt*. *n* = 4 mice, mean ± SEM. *P < 0.05 by unpaired *t*-test. (C) Protein level of IL-33 in the skin tissue of wild-type mice with indicated treatment for 24 h assessed by ELISA. *n* = 4, mean ± SEM. **P < 0.01 by unpaired *t*-test. (D and E) Representative immunofluorescent images showing the skin samples from *wild-type* mice with indicated treatment for 24 h. Quantification of IL-33 was shown in (E). *n* = 3, mean ± SEM. **P < 0.01 by unpaired *t*-test. (F) Expression of *Il33* in primary keratinocytes with indicated treatment for 12 h. Relative expression level was normalized to *Hprt*. *n* = 4, mean ± SEM. **P < 0.01 by unpaired *t*-test. (G) Protein level of IL-33 in primary keratinocytes and their supernatant with indicated treatment for 24 h assessed by ELISA. *n* = 3, mean ± SEM. **P < 0.01 by unpaired *t*-test. (H) Normalized peak tracks at the genomic locus of *Il33* identified by ATAC-Seq and Cut&Run-Seq. KC ATAC-Seq was performed on keratinocytes with indicated treatments. Tissue ATAC-Seq and KC Cut&Run-Seq were reanalyzed from a publicly available dataset (NCBI BioProject accession No. PRJNA731164). Dashed boxes indicate the five chromatin regions at the *Il33* locus that opened upon rhIL-24 treatment or wounding process (ATAC) and have STAT3 binding peaks (Cut&Run). KC, keratinocyte. (I) Binding of STAT3 to the genomic loci of *Il33* in cultured keratinocytes upon rhIL-24 treatment analyzed by ChIP-quantitative real-time PCR (ChIP-qPCR). (J, left) Expression of *Il33* in the skin of wild-type mice with indicated treatment for 12 h. Ruxolitinib (Rux) was used to block JAK-STAT signaling. Relative expression level was normalized to *Hprt*. *n* = 3 mice, mean ± SEM. *P < 0.05 by unpaired *t*-test. ns, not significant. (J, right) Protein level of IL-33 in the skin of wild-type mice with indicated treatment for 24 h assessed by ELISA. Ruxolitinib (Rux) was used to block JAK-STAT signaling. *n* = 8 mice, mean ± SEM. **P < 0.01, ***P < 0.001 by unpaired *t*-test. ns, not significant. (K) Expression of *Il33* in the skin from *Il20rbf*^*fl*/*fl*^ and *Krt14*^*Cre*^; *Il20rbf*^*fl*/*fl*^ mice with indicated treatment. *n* = 3 mice, mean ± SEM. *P < 0.05 by unpaired *t*-test. ns, not significant. (L) Proposed model of IL-24 function in exacerbating AD-like inflammation. KC, keratinocyte.

To further dissect the underlying mechanism by which IL-24 deteriorated AD-like disease, we performed RNA-Seq and profiled transcriptome at 8 h post rhIL-24 application on skin with vehicle group serving as control. KEGG pathway enrichment analysis showed the activation of JAK-STAT signaling pathway and Th1/Th2 cell differentiation (Fig. S9A). Moreover, heatmap and volcano plot identified that *Il33* was upregulated on transcriptional level, which was confirmed by RT-qPCR (Figs. S9B, S7A and S7B). Additionally, the expression of *Flg*, which encodes for the filaggrin (filament aggregating protein), was downregulated, together with the upregulation of *Tslp* and *Postn*, suggesting the potential role of barrier dysfunction caused by IL-24 (Fig. S9C). Further, when skin was treated with rhIL-24 for 24 h, IL-33 was increased in the tissue measured by ELISA (Fig. 7C). Accordingly, accumulation of IL-33 in the epidermis was verified by immunofluorescent staining (Fig. 7D and 7E). Consistently, the increased production of IL-33 was confirmed in primary keratinocytes both on transcription and protein level upon treatment with rhIL-24 (Fig. 7F and 7G).

The IL-33 content in the epidermis displayed a similar pattern of changes as was observed in STAT3 phosphorylation across the AD model groups (Fig. S9D and S9E). Therefore, we set to determine whether STAT3 signal could directly regulate *Il33* induction. Assay for transposase-accessible chromatin with high-throughput sequencing (ATAT-Seq) was performed on primary keratinocytes treated with vehicle or rhIL-24 to reveal the chromatin accessibility. Integrating the ATAC-Seq and STAT3 Cut&Run-Seq (cleavage under targets and release using nuclease-Seq) data during would healing from Liu et al. (2023), which shows that *Il24* was induced at wound edges upon wounding and hypoxia, we profiled regions containing STAT3 and STAT1 consensus DNA-recognition motif in ATAC-Seq by Homer *de novo* motif analysis (Fig. S9F). Importantly, we could locate STAT3 binding sites at the promoter and intergenic regions of *Il33* gene (Fig. 7H). The recruitment of STAT3 to the identified genomic regions was further confirmed by chromatin immunoprecipitation coupled with qPCR (ChIP-qPCR) (Fig. 7I). These data provide evidence for the regulatory function of IL-24-STAT in IL-33 induction upon AD-like disease development.

Lastly, we administered Ruxolitinib, a selective JAK inhibitor, via cutaneous delivery to assess whether blocking the JAK-STAT signaling pathway can prevent the increase of IL-33 induced by IL-24 stimulation. In Ruxolitinib-treated group, transcript analysis exhibited a reduction of *Il33* in response to IL-24 treatment, and ELISA analysis showed a decrease in IL-33 protein levels in tissue (Fig. 7J). Meanwhile, the specific deletion of *Il20rb* in keratinocytes abrogated the increased production of IL-33 (Figs. 7K, S9G and S9H). These findings suggest that IL-24 exacerbates AD-like disease conditions by promoting type 2 immunity through the JAK-STAT-IL-33 axis.

## Discussion

In this study, we found that IL-24 was induced in keratinocytes by MRSA infection and promoted type 2 immune response (illustrated in Fig. 7L). We observed that mice with *Il24*-deleted keratinocytes exhibited reduced tissue levels of IL-4 and IgE, as well as decreased circulating IgE in response to AD model induction. This was accompanied by enhanced skin barrier function and reduced itching. Consistently, the symptoms of asthma model, mimicking atopic march from epicutaneous sensitization, were relieved in *Il24*-deleted group. Conversely, cutaneous administration of recombinant IL-24 protein exacerbated type 2 immune reactions in the AD model. At the cellular level, IL-24 increased the production of IL-33 by keratinocytes through IL-20Rb paired receptor complexes, which in turn promoted allergic inflammation. These results demonstrate the critical role of IL-24 in the cytokine network involved in intercellular communication and the development of AD-like inflammation.

For AD management, topical corticosteroids (TCS) and topical calcineurin inhibitors (TCI) are currently adopted as the primary treatments (Freitas et al., 2022; Sideris et al., 2022). However, using these agents continuously and long-term raises concerns about adverse effects (Freitas et al., 2022; Sideris et al., 2022). While neutralization antibodies or inhibitors against IL-4Ra, IL-13, or JAK1/2 have been developed with some being approved (Bieber, 2022; Sideris et al., 2022), their efficacy and safety in the long run require comprehensive evaluation. Regardless of the availability of the current therapy, the inherent heterogeneity of AD itself can result in limited response rates (Bieber, 2022). Our research suggests that IL-24 has the potential to be a new therapeutic candidate for AD management. The inducible expression pattern of IL-24 indicates that it could be modulated by pharmacologic or microbial intervention. Additionally, its high expression in keratinocytes makes it suitable for topical intervention with minimal systemic side effects (Sideris et al., 2022). Blocking IL-24 or its receptor in the context of AD treatment represents a fresh and distinct approach.

IL-33 is a member of the alarmin group, which also includes TSLP and IL-25. These alarmins play a role in skin homeostasis and tissue repair, and at the same time lead to skin inflammation and diseases (Carmi-Levy et al., 2011; Hasegawa et al., 2022; Imai et al., 2013; Liu, 2006; Schmitz et al., 2005; Soumelis et al., 2002; Wang et al., 2007; Yoo et al., 2005). Our findings suggest that IL-24 can be induced to alert the disruption of tissue barrier in a similar manner. The responsive cells to IL-24 appear to be predominantly differentiated keratinocytes, unlike the aforementioned alarmins that signal primarily to immune cells. This suggests that IL-24 may serve as a messenger molecule to alter the expression profiles of differentiated keratinocytes that consequently drive a type 2 immune response due to their rapid cell death and increased amount of IL-33. Notably, other than the effects on wound healing observed on a receptor-dependent manner (He and Liang, 2010; Kolumam et al., 2017; Liu et al., 2023), IL-24 has been revealed in promoting tumor cell-specific apoptosis, maintaining Th17 homeostasis and ER homeostasis through a receptor-independent way (Chong et al., 2020; Sauane et al., 2004; Sie et al., 2022; Wang et al., 2020). Collectively, those findings suggest that the signaling pathways dictated by IL-24 can be diverse, and further investigations of its physiological functions are needed to fully understand how IL-24 coordinates tissue repair and skin immunity in various cellular settings (Rutz et al., 2014).

Patients with AD often suffer from other allergic conditions such as food allergy and asthma, and the underlying mechanism is not clear (Leyva-Castillo et al., 2019; Li et al., 2022; Paller et al., 2019). Reduced IgE in circulation from *K14*^*Cre*^; *Il24*^*fl*/*fl*^ animals suggest that skin-derived IL-24 may serve as a priming factor in accelerating allergic inflammation in distant organs, such as the lung and gut. Moreover, lacking a heparin-binding domain, IL-24 may escape from the extracellular matrix to the blood, which was evidenced by a recent finding that IL-20 subfamily cytokines increased significantly in the serum of patients with active eosinophilic esophagitis (EoE) (Kaymak et al., 2023), indicating the possibility of these cytokines functioning in an endocrine way. Interestingly, IL-24 is a common and specific autoantigen of IgE in patients with chronic spontaneous urticarial, highlighting a potentially broad implication of IL-24 in immune disorders (Schmetzer et al., 2018). Deng et al. recently reported that protease V8 from *S*. *aureus* activates protease-activated receptor-1 (PAR1) expression on sensory neurons to drive itch and skin damage (Deng et al., 2023). Re-analysis of scRNA-seq datasets from mouse and human sensory neurons showed the expression of *Il20ra* and *Il22ra1* were barely detectable in DRG (dorsal root ganglia) subsets, which was confirmed by RT-qPCR in mouse DRG, indicating IL-24 may not directly act on sensory neurons (Fig. S10). Nonetheless, as we continue to identify the immune mediators such as IL-24, new avenues will be incurred which will inform the inter-organ communications involved in the allergic comorbidities.

Our findings showed that MRSA infection resulted in immune changes in the skin and IL-24 was identified as a key MRSA-induced molecule promoting type 2 inflammation and AD-like disease severity. We have unraveled a crucial link between microbiota, skin cell adaptation and immune alteration in homeostasis and disease. A nexus is therefore established in the cytokine network to materialize the intercellular landmap in the skin. The work here has important implications for therapeutic development in AD treatment associated with different microbial colonization.

The level of IL-24 is heavily affected by *S*. *aureus* exposure in the skin rather than many other pathogens and allergens; however, our comprehension of IL-24 induction awaits further exploration. When infected with *S*. *aureus*, a range of virulence factors are produced (Tam and Torres, 2019; Thammavongsa et al., 2015), which could potentially play a role in *Il24* induction (Myles et al., 2013; Tam and Torres, 2019). Liu et al. recently elucidated that *Il24* expression depends on both IL-24/STAT3 signaling and hypoxia-stabilized hypoxia-inducible factor-1α (HIF1a) during wound healing in sterile injury (Liu et al., 2023). Early *in vitro* studies have shown that IL-24 may be positively regulated by epidermal inflammation in response to environmental and endogenous toxic stressors (Jin et al., 2014). Future investigation of IL-24 induction at the molecular level will reveal insights into how IL-24 is modulated by bacterial colonization versus sterile injury and a better understanding of the mechanisms of keratinocytes from innate immunity to adaptive responses.

## Materials and methods

### Study design

The objective of this study was to understand how *S*. *aureus*-host interplay exacerbates allergic inflammation and atopic dermatitis. We examined the transcriptional dynamics after MRSA infection, and identified the upregulation of *Il24*, as assessed by RNA-Seq, scRNA-Seq and RT-qPCR. Further, we found that *Il24* was produced by keratinocytes during MRSA infection, as evidenced by scRNA-Seq, HCR assay, and mass spectrometry. By genetic ablation of *Il24* and its receptor *Il20rb* in keratinocytes and cutaneous administration of recombinant IL-24 in a mouse model, we discovered that IL-24 promotes allergic inflammation and AD-like disease through JAK-STAT signaling, marked by dramatic changes in ear thickness, itching behavior, tissue IL-4 and IgE, circulating IgE, epidermal thickness and STAT phosphorylation. Moreover, by combining transcript analysis, protein assessment, RNA-Seq with a set of epigenomic sequencing analysis, we found that IL-24 exacerbates AD-like skin inflammation by enhancing IL-33 production, which could be blocked by JAK inhibitors.

### Mice

Mice were maintained on the 12 h/12 h light/dark cycle with the chow diet and water available ad libitum. The experimental procedures in mice were performed in compliance with the protocol approved by the Institutional Animal Care and Use Committee (IACUC) of Tsinghua University. The C57BL/6J wild-type and mutant mice were in-house bred to produce the littermates for experiments. Both male and female mice at 6–12 weeks old maintained in specific pathogen-free (SPF) conditions were utilized in the experiments.

*Krt14* ^*Cre*^ (JAX, 018964; RRID: IMSR_JAX: 018964) was from The Jackson Laboratory. *Il24*^*fl*/*fl*^ mice were generated with the targeting vector containing the loxP sites inserted to flank exon 2 of the *Il24* gene. The donor DNA plasmid was delivered together with Cas9 mRNA and single guide RNA (sgRNA) (5ʹ-sgRNA1: 5ʹ-GGTCAGGGTCTAAGAACCCCAGG-3ʹ; 3ʹ-sgRNA2: 5ʹ-GTTCTAGACTCAGGAGAAGGAGG-3ʹ) into C57BL/6J mouse zygotes via microinjection. The resulting offspring were screened by PCR genotyping and DNA sequencing to select the targeted mice. The mice were bred in-house to produce the littermates, which were randomly assigned to experimental groups. Likewise, *Il20rb*^*fl*/*fl*^ mice were generated by targeting exon 2 of the *Il20rb* gene. (5ʹ-sgRNA1: 5ʹ-TTGAACCTGATTCCCATTGGTGG-3ʹ; 3ʹ-sgRNA2: 5ʹ- AAGTTACCTGGTCATTGGAGAGG-3ʹ) into C57BL/6J mouse zygotes via microinjection.

### Atopic dermatitis and asthma model

Atopic dermatitis (AD) model was induced as previously described (Wang et al., 2021). In brief, 2 days before the induction (Day −2), the right cheek and right flank of upper-back skin were shaved. To induce chronic itch response, the exposed areas and bilateral ear skin were topically treated with 0.5 nmol of MC903 (calcipotriol, Tocris Bioscience) in 15 μL of 100% ethanol and then with 20 μL of 5 mg/mL ovalbumin (OVA, Macklin, China) in phosphate-buffered saline (PBS) daily for 10 days (Days 0–9). To provoke acute itch responses, mice were given an intradermal (i.d.) injection of 20 μL of 2.5 mg/mL OVA in 0.9% saline to the right cheek on Day 10. Scratching behavior was recorded from 60 min before i.d. OVA challenge (chronic spontaneous itch) to 70 min after the challenge (acute itch flare).

Asthma model was induced by adjusting the protocols previously reported (Segaud et al., 2022; Spergel et al., 1998) (Fig. S4A). Briefly, after epicutaneous sensitization, mice were intranasally (i.n.) treated with 50 μg OVA every two days. On Day 18, mice were recorded for at least 5 min by whole-body plethysmograph (WBP) system before and following 50 μg nebulized OVA inhalation. WBP analysis of Penh (enhanced pause) was calculated according to the equation: Penh (% change) = Penh (following inhalation)/ Penh (before inhalation) × 100%

### Bacterial infection

MRSA USA300 was a gift from Dr. Jing-ren Zhang (Tsinghua University). MRSA was streaked onto tryptic soy agar [tryptic soy broth (TSB) plus 1.5% agar; BD Biosciences] and single colonies were placed into TSB and grown overnight at 37°C in an incubator.

Mid-logarithmic phase bacteria were obtained after a 2 h subculture of a 1:50 dilution of the overnight culture. Bacterial concentrations were estimated by measuring the absorbance at 600 nm (A600) (Nanodrop; Thermo Scientific). Bacteria were pelleted, resuspended, and washed 3 times in PBS. After being heat-killed (65°C for 30 min) and lyophilized, HIMRSA was stored in −20°C. For HIMRSA treatment or recombinant protein injection during sensitization, as indicated in schematic diagram in figures, right cheek and right flank of upper-back skin were intradermally (i.d.) injected with HIMRSA (50 μg/lesion) or recombinant protein (100 ng/lesion; rhIL-24, SinoBiological; rmIL-24, R&D Systems) diluted in saline to a total volume of 20 μL per lesion.

### Behavioral tests

Behavioral tests were conducted as previously described (Wang et al., 2021). All applicable behavioral tests were performed and analyzed single-blindly. The optimal experimenting time to perform itch behavioral tests was between ZT0 and ZT12. To mock the effect of i.d. injections, animals were habituated in the test chamber for 90 min with a capped needle being pressed against the shaved cheek three times 2 days before recording. On the day of behavioral test, the experimental mouse was placed in the test chamber 10 min in advance to better acclimatization. To keep tabs on the number of scratching bouts during a 30-min period, video recordings were manually assessed. Of note, an instance of hind paw-dominated incessant scratching of the back, cheeks, or ears, which ended with the hind paw being placed in their mouth or to the chamber floor, was defined as one bout of scratching.

### Cell culture and stimulation

Primary murine keratinocytes were isolated from newborn pups (1 or 2 d after birth) and cultured in Medium 154CF (Gibco) supplemented with 0.05 mmol/L Ca^2+^ and human keratinocyte growth supplement (HKGS, Gibco). HEK293T cells were cultured in Dulbecco’s modified Eagle’s medium (DMEM, Corning) containing 10% fetal bovine serum (FBS, NEWZERUM), 100 U/mL of penicillin and streptomycin (Gibco) under standard culture conditions. All cells were cultured at 37°C and 5% CO_2_.

Keratinocyte culture was conducted according to previous studies with minor modifications (Li et al., 2017). In brief, skin tissue was dissected from *wild-type* neonates followed by digesting with 4 mg/mL of dispase II (Sigma-Aldrich) overnight at 4°C. The epidermis was then processed with TrypLE Express (Thermo Fisher Scientific) for 10 min at 37°C. Single-cell suspension was generated by vigorously rubbing epidermis tissue and passing fluid through a 100 μm filter. The cells were cultured in Medium 154CF supplemented with 0.05 mmol/L CaCl_2_ and HKGS at a density of 10^6^ cells/mL. To test keratinocytes in response to cytokines or pathogens, cells were stimulated by the indicated concentrations of cytokines or bacteria for the indicated time.

### Immune cell culture and flow cytometry

SiglecF^+^ eosinophils, SiglecF^−^ immune cells, CD11c^+^, CD3^+^, and CD11b^+^ cells were isolated from splenocytes and inguinal lymph node by magnetic cell sorting according to manufacturer’s instruction (Catalog No. 480080, BioLegend) using PE-anti-SiglecF (both positive and negative selection) (BD Biosciences; Cat# 552126; RRID: AB_394341), CD11c (BioLegend; Cat# 117347; RRID: AB_2563654), CD3ε (BioLegend; Cat# 100307; RRID: AB_312672), CD11b (BioLegend; Cat# 101207; RRID: AB_312790) antibodies respectively. For BMDC culture, freshly isolated bone marrow cells were cultured in 12-well plates at a density of 5 × 10^5^ cells/well in RPMI supplemented with recombinant murine GM-CSF (20 ng/mL) and IL-4 (10 ng/mL) (SinoBiological) as previously described (Inaba et al., 1992; Son et al., 2002). For naïve CD4 T cells culture, Immune cells from splenocytes and inguinal lymph node were labeled with PE/Cyanine7 anti-CD4 (BioLegend; Cat# 100407; RRID: AB_312692), FITC anti-CD44 (BioLegend; Cat# 103021; RRID: AB_493684) and PE anti-CD62L (BioLegend; Cat# 104407; RRID: AB_313094) antibodies and sorted by fluorescent-activated cell sorting (FACS; S3e Cell Sorter, Bio-Rad). Sorted cells were activated by anti-CD3 antibodies (BD Biosciences; Cat# 567114; RRID: AB_2916448) coated on plate and anti-CD28 antibodies (BD Biosciences; Cat# 567110; RRID: AB_2916447) in medium, and were induced to Type 2 helper T cells by adding with recombinant murine IL-4 protein and anti-IFNγ (BD Biosciences; Cat# 559065; RRID: AB_2123177)/anti-IL-12 (BD Biosciences; Cat# 554477; RRID: AB_398559) antibodies. Cells with indicated treatment were processed with intracellular (nuclear) labeling by anti-GATA3 antibody using Foxp3/Transcription Factor Staining Buffer Set (eBioscience). Samples were analyzed using LSR II (BD Biosciences) and FlowJo v10.8.1 software (BD Biosciences).

### Inflammation assessment and histopathology

Ear thickness was measured with digital calipers on Days 0 and 10 with it being an external manifest of mouse ear skin inflammation induced by MC903 + OVA. Mice were euthanized, and tissues were harvested for analysis after i.d. OVA challenge on Day 10 of the AD-associated acute itch flare model. The skins were fixed in 4% paraformaldehyde (PFA; Thermo Fisher Scientific) and processed for paraffin-sectioning and H&E (hematoxylin and eosin) staining. Slides were imaged using Axio Scan Z1 (Zeiss). For asthma model, on Day 18, lung tissues were harvested for analysis after WBP. Tissues were fixed in 4% PFA and then dehydrated with 30% glucose for cryo-section (Leica).

### Immunofluorescent staining

The mice were euthanized at indicated time points post-treatment or onset of AD, and indicated tissues were harvested. For the immunofluorescent staining, dissected ears or skins were immediately embedded in optimal cutting temperature (OCT) medium (Sakura) and sectioned at 10 μm on a cryotome (Leica). Then, sections were fixed in 4% PFA (Thermo Fisher Scientific) at room temperature for 10 min followed by dehydration with ethanol from 50%, 75%, 100%, and 5 min for each by the gradient. Dehydrated sections were immunolabeled with indicated primary antibodies and corresponding Alexa dye-conjugated secondary antibodies and imaged using fluorescence microscopy (Nikon). Specifically, skin from p0 mice was treated *ex vivo* with rhIL-24 in Medium 154CF supplemented with 0.05 mmol/L Ca^2+^ and HKGS as mentioned above, followed by the staining procedures. Lung tissues from the asthma model were dehydrated before sectioning. Primary antibodies include: rabbit anti-p-STAT1 (Cell Signaling Technology; Cat# 9167; RRID: AB_561284), rabbit anti-p-STAT3 (Cell Signaling Technology; Cat# 9145; RRID: AB_2491009), mouse antikeratin 14 (Santa Cruz Biotechnology; Cat# sc-53253; RRID: AB_2134820), mouse antikeratin 10 (Santa Cruz Biotechnology; Cat# sc-23877; RRID: AB_2134668), goat antimouse IL-33 (R&D Systems; Cat# AF3626; RRID: AB_884269), rat PE-anti-SiglecF (BD Biosciences; Cat# 552126; RRID: AB_394341).

### RT-qPCR

Mouse skin tissues (5 mm in diameter) were homogenized in TRIzol using a hand-hold homogenizer, whereas cells were directly lysed and resuspended in TRIzol. The total RNA of dissected tissue or cells was extracted by RNeasy Mini Kit (QIAGEN) followed by qPCR analysis (2× M5 HiPer SYBR Premix Es Taq (Mei5bio, Beijing, China)) on Step One Plus Real-Time PCR System (Applied Biosystems) with two technical replicates using primers listed in List of RT-qPCR primers (Supplementary Materials). Results were normalized to *Hprt* (Hypoxanthine guanine phosphoribosyl transferase) and the comparative ∆∆CT method was used to determine the quantification of gene expression.

### ELISA analysis

For measurement of IgE in serum by ELISA, whole blood was collected into 1.5 mL microcentrifuge tubes and allowed to clot for 30 min at room temperature. After centrifugation at 200 ×*g* for 10 min in a refrigerated centrifuge, the supernatant was collected and measured by the IgE ELISA kit (Catalog No. 432404; BioLegend) with 100–200-fold dilution. The sensitivity of the kit is 0.1 ng/mL, and the standard range is 0–10 ng/mL.

For the measurement of IgE and IL-4 in tissues by ELISA, the mice were scarified by cervical dislocation. Right cheeks or lung tissues [tissue weight (TW) was measured, approx. 30 mg for each sample] were dissected, followed by homogenization in 2 mL extraction buffer [100 mmol/L Tris-HCl (pH 7.4), 150 mmol/L NaCl, 1 mmol/L ethylene glycol tetraacetic acid (EGTA), 1 mmol/L EDTA, 1% Triton X-100, 0.5% sodium deoxycholate, protease inhibitor cocktail (TargetMol), and 1 mmol/L phenylmethylsulfonyl fluoride (PMSF)]. The samples were then centrifuged for 20 min at 13,000 rpm at 4°C to remove cell debris and lipids. The samples were then applied to measurement using an IgE ELISA kit (Catalog No. 432404; BioLegend) and IL-4 ELISA kit (Catalog No. 431104; BioLegend). The concentrations were calculated according to the instructions from the manufacturer. Data were presented following equation: Conc. [ng(IgE)/g or pg(IL-4)/g] = concentration (ng/mL or pg/mL) × 2 mL/TW(g).

### Immunoblotting

The pieces of skin (5-mm in diameter) were homogenized in precooled radioimmunoprecipitation (RIPA) buffer, pH 7.4, containing protease inhibitor cocktail (TargetMol), whereas cells with different treatments were lysed directly. A total of 20 μg protein was subjected to sodium dodecyl-sulfate (SDS)-polyacrylamide gel electrophoresis (PAGE) and blotted using the indicated antibodies. Primary antibodies include rabbit anti-p-STAT1 (Cell Signaling Technology; Cat# 9167; RRID: AB_561284), rabbit anti-p-STAT3 (Cell Signaling Technology; Cat# 9145; RRID: AB_2491009), rabbit anti-STAT3 (Cell Signaling Technology; Cat# 12640; RRID: AB_2629499), rabbit anti-GAPDH (Cell Signaling Technology; Cat# 5174; RRID: AB_10622025), rabbit anti-β-Actin (BioLegend; Cat# 622102; RRID: AB_315946), mouse antikeratin 14 (Santa Cruz Biotechnology; Cat# sc-53253; RRID: AB_2134820).

### Mass spectrometry

Supernatant from HIMRSA/HBSS-treated primary keratinocytes was collected and concentrated using Pierce Concentrator (Thermo Fisher Scientific). For knockout verification, skin tissues from MRSA-infected *Il24*^*fl*/*fl*^/*Krt14*^*Cre*^; *Il24*^*fl*/*fl*^ mice were collected. Then, 10 μg of total protein was subjected to SDS-PAGE. After Coomassie Brilliant Blue staining, bands between 15–35 kDa were excised. In-gel digestion and tandem mass spectrometry analysis were performed by the Protein Chemistry and Omics Platform, Tsinghua University. Briefly, the sample was digested in SDS gel and analyzed on LC-MS. The peptide/spectrum matches (PSMs) and area were evaluated for candidate choice. IL-24 protein sequence (UniProt ID: Q925S4) was searched against MS spectra. Specific peptide sequence “IVIMSQLQPSKDNSMLPISESAHQRFLL” (positions in protein: 121–146) was used to quantify abundance.

### Transient transfection and luciferase reporter assay

The coding sequences (CDS) of mouse IL-20R subunits (mouse *Il20ra*, *Il20rb* and *Il22ra1)* were cloned to pGCMV-IRES-GFP plasmids. The plasmids encoding various IL-20R subunits were transiently transfected into HEK293T cells in different combinations using polyethyleneimine (PEI) according to the manufacturer’s recommendations. Cells were stimulated with indicated cytokines for the indicated time at 36 h post transfection and then were collected for immunoblotting. The reporter plasmid pGL4.26-4 × m67-SIE-Luciferase was constructed with four copies of the m67-SIE responsive element for STAT1/3 inserted into the pGL4.26 Vector (Promega) (Bromberg et al., 1999). For luciferase reporter assay, apart from the plasmids encoding IL-20R described above, indicated luciferase reporter plasmids and internal control pCMV-Renilla plasmids were transfected into cells at the same time. Thirty-six hours after transfection, cells were stimulated with indicated cytokines for 12 h, and then were lysed and analyzed with Dual-Luciferase Reporter Assay System (Beyotime, Shanghai).

### *In situ* hybridization chain reaction (HCR) experiment

Hybridization probes were designed to target *Il24* mRNA (NCBI Reference Sequence: NM_053095.3). All HCR probes for detection were synthesized by Sangon Biotech Inc. with PAGE purification. Fluorescent hairpins were synthesized by Sangon Biotech Inc. with HPLC purification. DNA sequences were listed in the list of HCR probes and hairpins (Supplementary Materials).

*In situ* hybridization chain reaction (HCR 3.0) staining of primary keratinocytes was done following the published protocols (Choi et al., 2018). In detail, primary keratinocytes were cultured and treated with HIMRSA. Cells on a chambered slide were fixed with 4% PFA and permeabilized in ice-cold 70% ethanol at −20°C overnight. Cell samples were then prehybridized with HCR probe hybridization buffer for 30 min at 37°C. During prehybridization, HCR probes were diluted in the hybridization buffer (final concentration of each probe is 4 nmol/L). After prehybridization, discard the buffer from cell samples and reload the 300 μL probe solution. Cell samples were incubated with probes overnight at 37°C. After hybridization, cell samples were washed with HCR probe wash buffer for 4 × 5 min at 37°C to remove excess probes and then underwent washing with 5× SSCT for 2 × 5 min at room temperature. For *in situ* HCR staining of the skin tissue, cryo-sections of the tissue were processed as reported (May-Zhang et al., 2022). Basically, after prefixed with 4% PFA and dehydrated in ethanol from 50%, 75%, 100%, and 5 min for each by the gradient at RT. Dehydrated sections were frozen at −80°C for at least 24 h, followed by fixation with 4% PFA and proteinase K (NEB) treatment. Hybridization was performed as mentioned above.

For signal amplification, samples were preincubated with an HCR amplification buffer for 30 min at room temperature. Fluorescent hairpins were annealed separately with the following annealing protocol: 95°C for 1.5 min and then naturally cooled to room temperature. Annealed hairpins were diluted in amplification buffer (final concentration of each probe is 60 nmol/L). Cell samples were incubated with hairpins overnight in the dark at room temperature and then washed with 5× SSCT for 6 × 5 min to remove excess hairpins. After washing, Fluoromount-G mounting medium with DAPI was added to the samples. The samples were imaged using fluorescence microscopy (Nikon).

### RNA-Seq and data analysis

The total RNA was extracted and processed for RNA sequencing. The RNA sequencing data were mapped to the mouse reference genome by HISAT2/Bowtie2 tool. The differential expression genes upregulated or downregulated by 2-fold or higher were analyzed further, with the adjusted *P* value (padj) less than 0.05.

### Assay for transposase accessible chromatin with high-throughput sequencing (ATAC-Seq) and data analysis

ATAC-Seq was performed on primary keratinocytes from rhIL-24 and vehicle control treatment and processed according to the manufacturer’s instruction (Vazyme). Briefly, treated cells were lysed with lysis buffer for 10 min [10 mmol/L Tris-HCl (pH 7.4), 10 mmol/L NaCl, 3 mmol/L MgCl_2_, 0.5% NP-40] as previously described (Wu et al., 2018). Lysed cells were transposed with TN transposase (Catalog No. TD501; Vazyme) for 18 min followed by DNA purification, barcoding, and library sorting (Catalog No. TD202; Vazyme). Libraries were sequenced on Illumina. Paired-end ATAC-Seq and Cut&Run-Seq FASTQs were processed with TrimGalore (v0.6.7) for quality control and further aligned to the mm10 genome using Bowtie2 (v2.3.5.1) (Langmead and Salzberg, 2012). Peak calls for each sample were made with MACS2 software (Zhang et al., 2008). The *de novo* motif analysis was carried out by HOMER (Heinz et al., 2010).

### Chromatin immunoprecipitation coupled with quantitative real-time PCR (ChIP-qPCR)

To determine the target genes of transcription factor STAT3, ChIP experiment was carried out. ChIP experiment was done following instructions of SimpleChIP Plus Enzymatic Chromatin IP Kit (Cell Signaling Technology, #9005). Chromatin was prepared from 8 × 10^6^ keratinocytes at 1 h after treatment with 100 ng/mL rhIL-24 or HBSS. Processed chromatin was immunoprecipitated by rabbit anti-p-STAT3 (Cell Signaling Technology, #9145) antibody. For the following quantitative real-time PCR, ChIP DNA input and DNA products were analyzed with 2× M5 HiPer SYBR Premix Es Taq (Mei5bio, Beijing, China) on Step One Plus Real-Time PCR System (Applied Biosystems) using primers listed in List of ChIP-qPCR primers (Supplementary Materials). Results were normalized to DNA input samples and the comparative ∆∆CT method was used to determine the quantification of the enrichment.

### Skin tissue dissociation and single-cell suspension preparation

Six pieces of lesional skin samples (5-mm in diameter) from mice challenged with MRSA or vehicle control for 8 h were harvested for scRNA-Seq. In general, skin samples were cut into small pieces (about 0.5 mm^2^) and digested with 0.25% Trypsin (Thermo Fisher Scientific; 37°C for 30 min) and followed by collagenase I (Sigma-Aldrich; collagenase I (1 mg/mL) and DNase I (100 μg/mL) in DMEM; 37°C for 30 min). Then 100 μm cell strainer was used to filter.

The cell suspension was centrifuged at 300 rpm at 4°C for 5 min. After washing with precooled PBS twice, cells were added with 3 mL of precooled erythrocytes lysis liquid (Solarbio) and dispersed evenly. Cells were incubated at 4°C for 5 min, centrifuged at 300 rpm at 4°C for 5 min immediately after completion and the supernatant was discarded. Cells were added with 1 mL of precooled PBS to fully re-suspend. When testing with a cell counter (Countstar Rigel S2), cell concentration was adjusted accordingly, with the target concentration being 700–1,200 cell/μL, the cell activity being greater than 90%, and the agglomeration being less than 15%. Once the final cell concentration and activity were reached, the cells were placed on ice and 10× Genomics single-cell transcriptome chip-on-board experiment was carried out within 30 min.

### Single-cell RNA-Seq library preparation and sequencing

Single-cell RNA-Seq libraries were prepared with Chromium Next GEM Single Cell 3ʹ Reagent Kits v3.1 on the Chromium Controller (10× Genomics). The cell suspension was loaded onto the Chromium Next GEM Chip G and ran the Chromium Controller to generate single-cell gel beads in the emulsion (GEM) according to the manufacturer’s recommendation. Captured cells were lysed and the released RNA was barcoded through reverse transcription in individual GEMs. Barcoded, full-length cDNA was generated, and libraries were constructed. The quality of libraries was assessed by Qubit 4.0 and the Agilent 2100. Sequencing was performed on the Illumina NovaSeq 6000 with a sequencing depth of at least 50,000 reads per cell and 150 bp (PE150) paired-end reads (performed by Biomarker Technologies Corporation, Beijing, China).

### Data analysis by single-cell RNA sequencing

We performed alignment to this amended reference using 10× cellranger (version 6.0.0), which employs the STAR sequence aligner (Dobin et al., 2013). The reference genome was the mouse genome mm10. Overall, 21,267 cells passed the quality control. To remove the cells with low quality, cells with gene numbers from 500 to 7,000 and the ratio of mitochondria lower than 20% were maintained, and genes with at least one feature count in more than 10 cells were used for the following analysis. We determined gene expression counts using unique molecular identifiers (UMIs) for each cell barcode-gene combination. Following alignment, we filtered cell barcodes to identify those that contain cells using the approach implemented in cell ranger (version 6.0.0), and only these barcodes were considered for downstream analysis including clustering and cell type identification, differential expression analysis by Seurat (version 4.0.1).

### Dimensionality reduction

To enable unsupervised clustering and cell type identification, we performed dimensionality reduction with principal component analysis (PCA) on the combined set of samples for each tissue. To visualize the data, we further reduced the dimensionality of all 21,267 cells using Seurat (version 4.0.1) and used t-SNE to project the cells into 2-D space. The steps include 1. Using the LogNormalize method of the “Normalization” function of the Seurat software to calculate the expression value of genes; 2. PCA analysis was performed using the normalized expression value. Within all the PCs, the top 10 PCs were used to do clustering and t-SNE analysis; 3. To find clusters, the weighted Shared Nearest Neighbor (SNN) graph-based clustering method was utilized. Marker genes for each cluster were identified by the “bimod” (Likelihood-ratio test) with default parameters via the FindAllMarkers function in Seurat. Filter |Fold Change| > 1.5 and FDR < 0.1 from the calculation results of find marker, and then sort to top10 gene as the marker gene.

### Clustering and cell type identification

We applied Louvain community detection (Satija et al., 2015) to the nearest neighbor graph constructed in PCA space to define a cluster partition. Specific top marker genes for each cell cluster are shown in Fig. S2B. We used singleR (version 4.0.4), which was based on Spearman correlation analysis, containing seven datasets to automate the identification of cell types. Specifically, according to the relative expression score to indicated cell types in the datasets, singleR annotate the corresponding cell types as shown in Fig. S2C. Top 10 feature genes of each cell type are listed in Table S1.

### Gene Ontology enrichment analysis

Enrichr (Kuleshov et al., 2016) was used to perform gene set enrichment analysis against the Gene Ontology Biological Process 2018 version gene set collection. We also used the MSigDB Hallmark gene sets (Liberzon et al., 2015), for which we computed enrichment scores using Fisher’s exact test. In both cases, we corrected for multiple hypothesis testing using the Benjamini–Hochberg procedure.

### Statistical analysis

Statistical analysis was calculated using Prism software (GraphPad). Unpaired two-tailed Student’s *t*-tests were used to assess the statistical significance between the two groups. Welch’s correction was used when the variances of the samples were unequal. The sample capacity can be found in the figure legends. Each “*n*” represents the number of mice or cell replicates and indicated in the figure legends. *P* < 0.05 was considered significant. *P < 0.05, **P < 0.01, ***P < 0.001, ****P < 0.0001, ns, not significant. Error Bars represent mean ± SEM.

## Supplementary data

The online version contains supplementary material available at https://doi.org/10.1093/procel/pwae030.

pwae030_suppl_Supplementary_Video_S1

pwae030_suppl_Supplementary_Figures_S1-S70_Tables_S1

pwae030_suppl_Supplementary_Video_S2

pwae030_suppl_Supplementary_Video_S3

## Data Availability

The raw sequence data reported in the present paper have been deposited in the Sequence Read Archive (SRA) database with accession number PRJNA975302 (Bulk RNA-Seq), PRJNA975496 (scRNA-Seq), PRJNA992643 (ATAC-Seq). The MS proteomics data have been deposited in the ProteomeXchange Consortium with accession number PXD042452 and PXD042453.

## Code availability

All the custom codes are available from the corresponding authors upon reasonable request.
