# Supplementary material for: IL-24 promotes atopic dermatitis-like inflammation through driving MRSA-induced allergic responses
Source: Protein Cell. 2024 May 16;16(3):188–210. doi: 10.1093/procel/pwae030 (PMC11892005; doi:10.1093/procel/pwae030)
Supplement: pwae030_suppl_Supplementary_Figures_S1-S70_Tables_S1 [file pwae030_suppl_supplementary_figures_s1-s70_tables_s1.pdf]

Supplementary Materials

IL-24 promotes atopic dermatitis-like inflammation through driving MRSA-induced allergic responses

Xinmin Qian, Meiyi Tong, Tianqing Zhang, Qingqing Li, Meng Hua, Nan Zhou, Wenwen Zeng

Figures

Fig. S1. Skin MRSA application induces *Il24* expression, related to Fig. 1.

A, KEGG pathway analysis of shared DEGs in skin samples from HIMRSA-treated group (24 hr) and vehicle-treated control group by RNA-Seq.

B, GO analysis in biological process of shared DEGs in skin samples from HIMRSA-treated group (8 hr) and vehicle-treated control group by RNA-Seq.

C, GO analysis in biological process of shared DEGs in lesional skin samples from 38 healthy controls and 27 patients with AD in RNA-Seq datasets (GEO database, accession no. GSE121212).

D, Heatmap showing the feature genes related to infection and allergic responses in skin samples from HIMRSA-treated group (8/24 hr) and vehicle-treated control group by RNA-Seq analysis.

E, Expression of *Il24* in MRSA-treated and HIMRSA-treated skin of *wild-type* mice at 8 hr post infection; results were presented by fold normalized to vehicle control. n= 5-8 mice per dose, mean ±SEM. * $p < 0.05$, ** $p < 0.01$ by unpaired t test.

F, Expression of *Il24* of the skin samples from *wild-type* mice with indicated challenges at 8 hr post treatment; results were presented by fold normalized to vehicle control. n= 3-4 mice, mean ±SEM. n.s., not significant by unpaired t test.

G, Morphology and H&E staining of the MRSA-infected lesional sites assessed from day 0 to day 15. Dashed lines indicate lesional sites.

H, Expression of *Cxcl1*, *S100a8*, *Defb4*, *Serpinb3a* and *Postn* in MRSA-treated skin of *wild-type* mice over the course of infection; results were presented by fold normalized to uninfected mice. n= 9-12 mice per time point, mean ±SEM. T.p.i., time post infection.

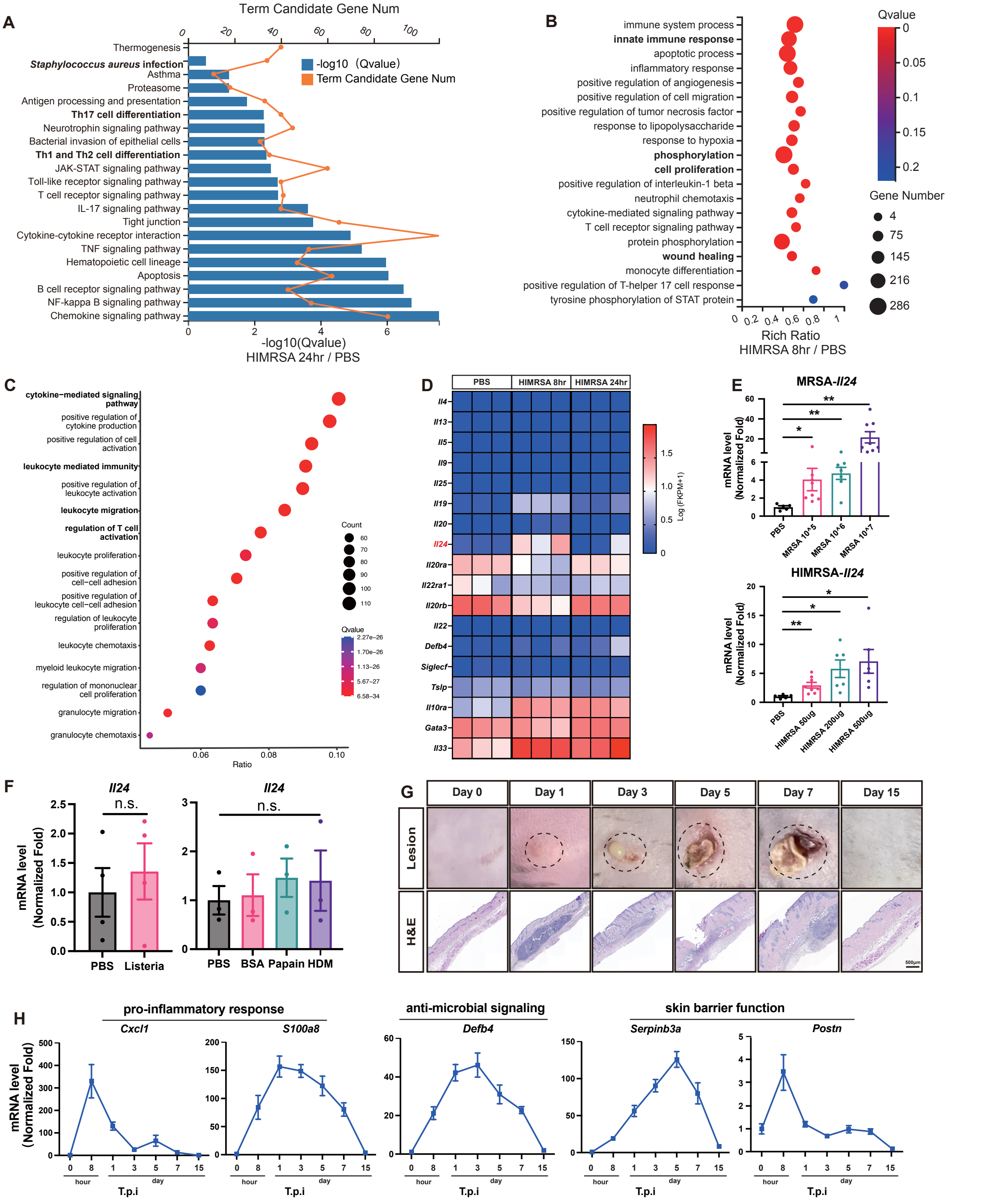

A
Term Candidate Gene Num
-log10 (Qvalue)
Term Candidate Gene Num
Thermogenesis
Staphylococcus aureus infection
Asthma
Proteasome
Antigen processing and presentation
Th17 cell differentiation
Neurotrophin signaling pathway
Bacterial invasion of epithelial cells
Th1 and Th2 cell differentiation
JAK-STAT signaling pathway
Toll-like receptor signaling pathway
T cell receptor signaling pathway
IL-17 signaling pathway
Tight junction
Cytokine-cytokine receptor interaction
TNF signaling pathway
Hematopoietic cell lineage
Apoptosis
B cell receptor signaling pathway
NF-kappa B signaling pathway
Chemokine signaling pathway
-log10(Qvalue)
HIMRSA 24hr / PBS
B
immune system process
innate immune response
apoptotic process
inflammatory response
positive regulation of angiogenesis
positive regulation of cell migration
positive regulation of tumor necrosis factor
response to lipopolysaccharide
response to hypoxia
phosphorylation
cell proliferation
positive regulation of interleukin-1 beta
neutrophil chemotaxis
cytokine-mediated signaling pathway
T cell receptor signaling pathway
protein phosphorylation
wound healing
monocyte differentiation
positive regulation of T-helper 17 cell response
tyrosine phosphorylation of STAT protein
Qvalue
Gene Number
Rich Ratio
HIMRSA 8hr / PBS
C
cytokine−mediated signaling pathway
positive regulation of cytokine production
positive regulation of cell activation
leukocyte mediated immunity
positive regulation of leukocyte activation
leukocyte migration
regulation of T cell activation
leukocyte proliferation
positive regulation of cell−cell adhesion
positive regulation of leukocyte cell−cell adhesion
regulation of leukocyte proliferation
leukocyte chemotaxis
myeloid leukocyte migration
regulation of mononuclear cell proliferation
granulocyte migration
granulocyte chemotaxis
Count
Qvalue
Ratio
D
PBS
HIMRSA 8hr
HIMRSA 24hr
Il4
Il13
Il5
Il9
Il25
Il19
Il20
Il24
Il20ra
Il22ra1
Il20rb
Il22
Defb4
Siglecf
Tslp
Il10ra
Gata3
Il33
Log (FKPM+1)
E
MRSA-Il24
mRNA level (Normalized Fold)
PBS
MRSA 10^5
MRSA 10^6
MRSA 10^7
HIMRSA-Il24
PBS
HIMRSA 50ug
HIMRSA 200ug
HIMRSA 500ug
F
Il24
n.s.
mRNA level (Normalized Fold)
PBS
Listeria
Il24
n.s.
PBS
BSA
Papain
HDM
G
Day 0
Day 1
Day 3
Day 5
Day 7
Day 15
Lesion
H&E
500μm
H
pro-inflammatory response
anti-microbial signaling
skin barrier function
Cxcl1
S100a8
Defb4
Serpinb3a
Postn
mRNA level (Normalized Fold)
hour
day
T.p.i

Fig. S2. Keratinocytes are the main producer of IL-24 upon MRSA infection, related to Fig. 2.

A, t-SNE plot displaying all 17 clusters of cells from mouse skin.

B, Dot plot showing the expression of specific top marker genes for each cell cluster.

C, Heatmap plot displaying the expression scores of 17 clusters corresponding to the indicated cell types.

D, Frequency of cells from skin of mice with MRSA and vehicle application.

E, Heatmap plot displaying feature genes related to infection and allergic responses for each cell type.

F, Correlation plots of mRNA levels of IL-24 receptor genes (**upper**); two typical type 2 cytokine genes (**bottom**) and *Krt14* across different cell types. Scatterplots with estimated linear regression and 95% confidence interval; R, Pearson correlation.

G and H, Expression of *Il24* (**G**) and *Krt14* (**H**) in different skin layers post HIMRSA application. Relative expression level was normalized to *Hprt*. n= 3, mean ± SEM.

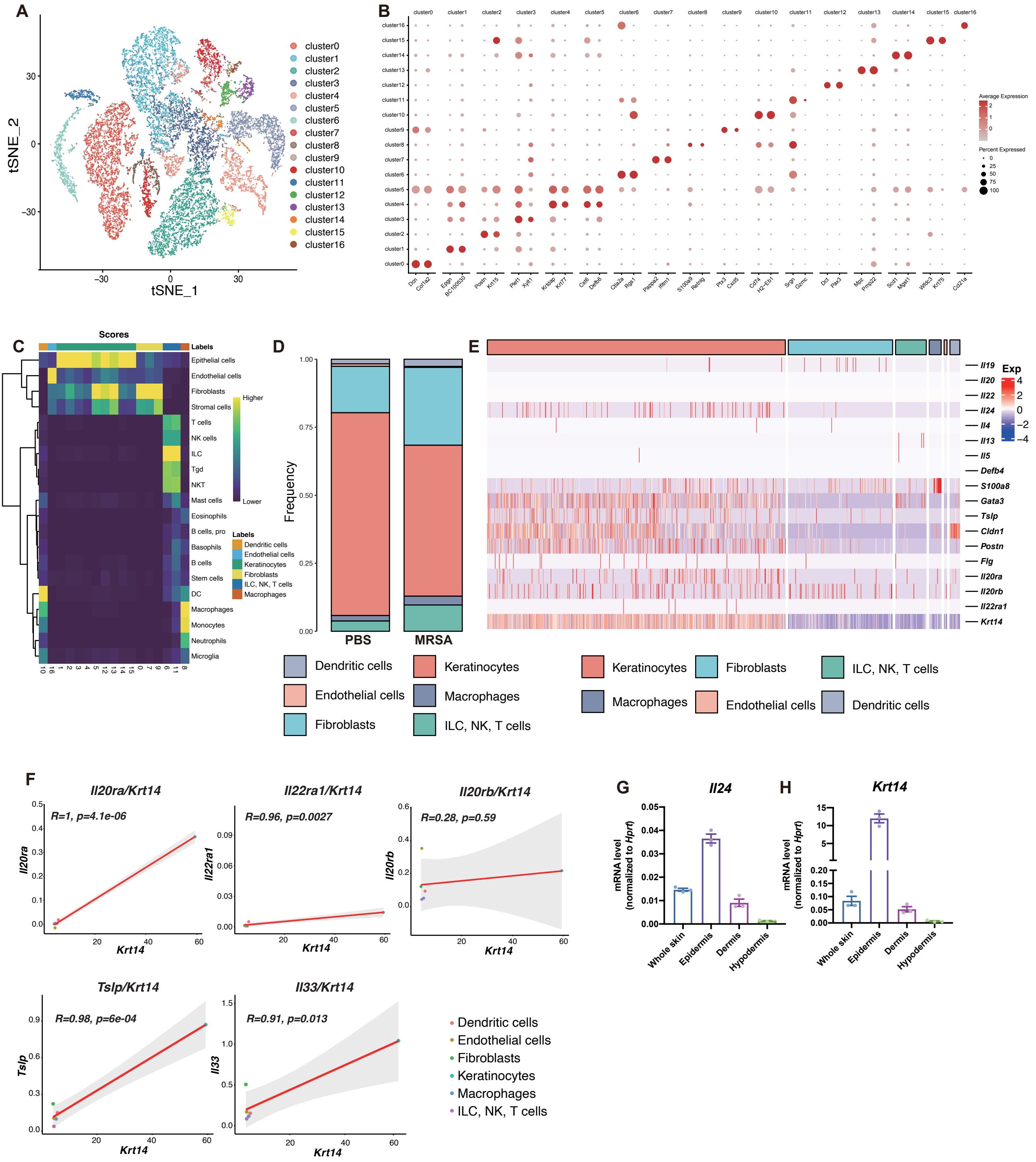

A
tSNE_2
tSNE_1
cluster0
cluster1
cluster2
cluster3
cluster4
cluster5
cluster6
cluster7
cluster8
cluster9
cluster10
cluster11
cluster12
cluster13
cluster14
cluster15
cluster16
B
Average Expression
Percent Expressed
Dcn
Col1a2
Epgn
BC100530
Postn
Krt15
Plet1
Xylt1
Krtdap
Krt77
Cst6
Defb6
Clda2a
Rgs1
Pappa2
Ifitm1
S100a9
Retnlg
Ptx3
Cxcl5
Cd74
H2-Eb1
Srgn
Gzmc
Dct
Pax3
Mpz
Pmp22
Scd1
Mgst1
Wfdc3
Krt75
Ccl21a
C
Scores
Labels
Epithelial cells
Endothelial cells
Fibroblasts
Stromal cells
T cells
NK cells
ILC
Tgd
NKT
Mast cells
Eosinophils
B cells, pro
Basophils
B cells
Stem cells
DC
Macrophages
Monocytes
Neutrophils
Microglia
Higher
Lower
Dendritic cells
Keratinocytes
ILC, NK, T cells
D
Frequency
PBS
MRSA
Dendritic cells
Keratinocytes
Endothelial cells
Macrophages
Fibroblasts
ILC, NK, T cells
E
Exp
Il19
Il20
Il22
Il24
Il4
Il13
Il5
Defb4
S100a8
Gata3
Tslp
Cldn1
Postn
Flg
Il20ra
Il20rb
Il22ra1
Krt14
Keratinocytes
Fibroblasts
ILC, NK, T cells
Macrophages
Endothelial cells
Dendritic cells
F
Il20ra/Krt14
R=1, p=4.1e-06
Il22ra1/Krt14
R=0.96, p=0.0027
Il20rb/Krt14
R=0.28, p=0.59
Tslp/Krt14
R=0.98, p=6e-04
Il33/Krt14
R=0.91, p=0.013
Krt14
Dendritic cells
Endothelial cells
Fibroblasts
Keratinocytes
Macrophages
ILC, NK, T cells
G
Il24
mRNA level (normalized to Hprt)
Whole skin
Epidermis
Dermis
Hypodermis
H
Krt14

Fig. S3. Keratinocyte-derived IL-24 drives AD-like inflammation, related to Fig. 3.

A, Schematic graph showing the strategy for generating *Krt14*^{Cre}; *Il24*$^{fl/fl}$ conditional knockout mice.

B and C, Deletion of *Il24* exon 2 in keratinocytes in *Krt14*^{Cre}; *Il24f*$^{fl/fl}$ mice, determined by RT-qPCR (**B**) and mass-spectrometry (**C**). n=3 mice, mean ± SEM. *p < 0.05, **p< 0.01 by unpaired t test.

D, Appearance of skin and hair in *Krt14*^{Cre}; *Il24*$^{fl/fl}$ mice and littermate control.

E and F, Representative immunofluorescent images showing the skin of *Krt14*^{Cre}; *Il24f*$^{fl/fl}$ mice and littermate control at steady state. Anti-keratin 14 antibody was used to label K14^{+} keratinocytes. The epidermal thickness was assessed and quantified in **F**. n= 4 mice, mean ± SEM. n.s., not significant by unpaired t test. Dashed lines indicate the border of epidermis.

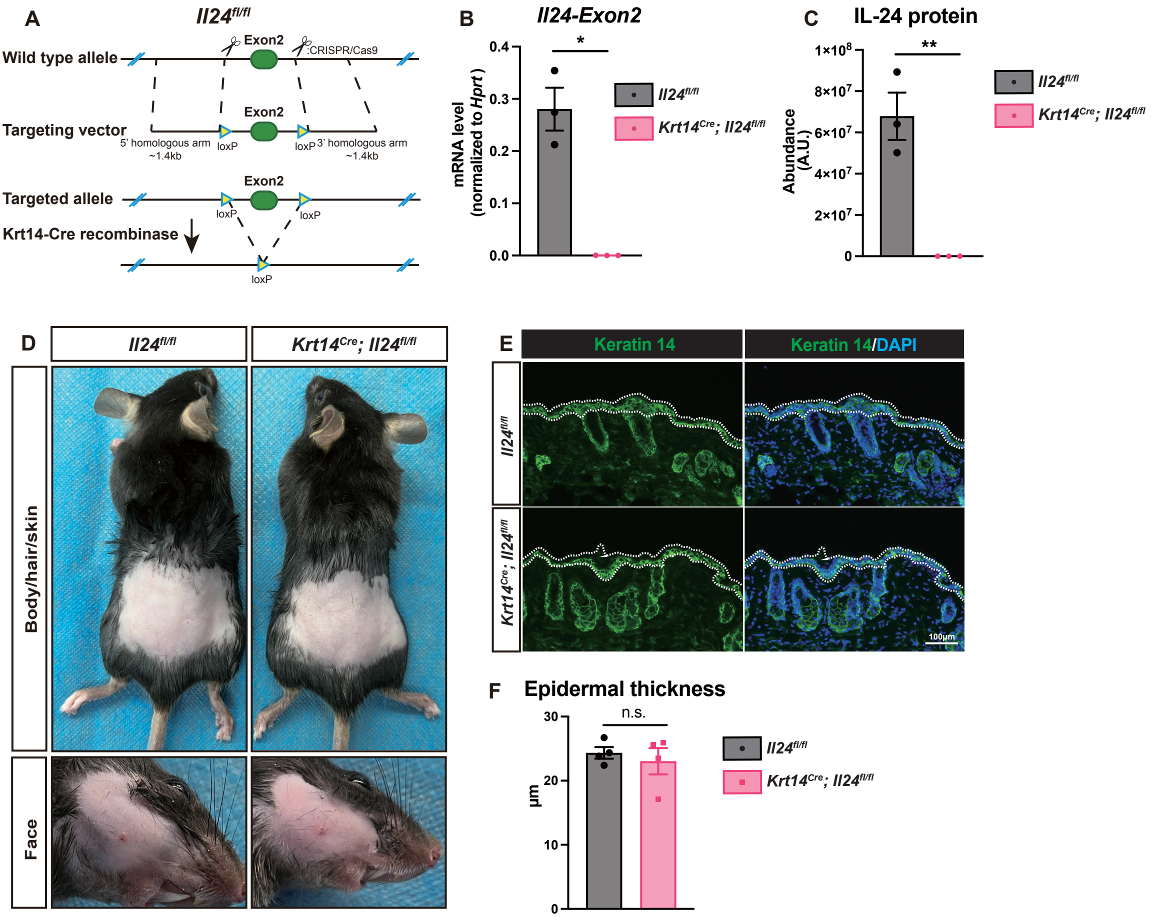
A
Il24fl/fl
Wild type allele
Exon2
:CRISPR/Cas9
Targeting vector
5' homologous arm ~1.4kb
loxP
loxP
3' homologous arm ~1.4kb
Targeted allele
Krt14-Cre recombinase
B
Il24-Exon2
mRNA level (normalized to Hprt)
C
IL-24 protein
Abundance (A.U.)
Il24fl/fl
Krt14Cre; Il24fl/fl
D
Body/hair/skin
Face
E
Keratin 14
Keratin 14/DAPI
100μm
F
Epidermal thickness
n.s.
μm

Fig. S4. Keratinocyte-derived IL-24 drives asthma-like disease conditions from epicutaneous sensitization, related to Fig. 3.

A, Schematic of mouse asthma-like disease model (OVA) and experimental pipeline. During sensitization stage, mice were intradermally (i.d.) challenged with HIMRSA every three days. During march stage, mice were intranasally (i.n.) challenged with OVA every two days.

B-D, Serum OVA-specific IgE (**B**), lung OVA-specific IgE (**C**), and lung IL-4 (**D**) in $Il24^{fl/fl}$ and $Krt14^{Cre}$; $Il24^{fl/fl}$ mice at steady state and after model induction. n= 3-7 mice, mean ± SEM. *$p < 0.05$ by unpaired t test.

E, Whole-body plethysmograph (WBP) analysis of Penh (Enhanced pause) was used to assess airway constriction. Nebulized OVA was inhaled by mice to induce constriction. n= 3-4 mice, mean ± SEM. *$p < 0.05$ by unpaired t test.

F and G, Representative immunofluorescent images showing the lung of $Il24^{fl/fl}$ and $Krt14^{Cre}$; $Il24^{fl/fl}$ mice at steady state or on day 18 post induction. Anti-SiglecF antibody was used to label $SigelecF^{+}$ cells, which were quantified in **G**. n= 3-6 mice, mean ± SEM. **$p < 0.01$ by unpaired t test.

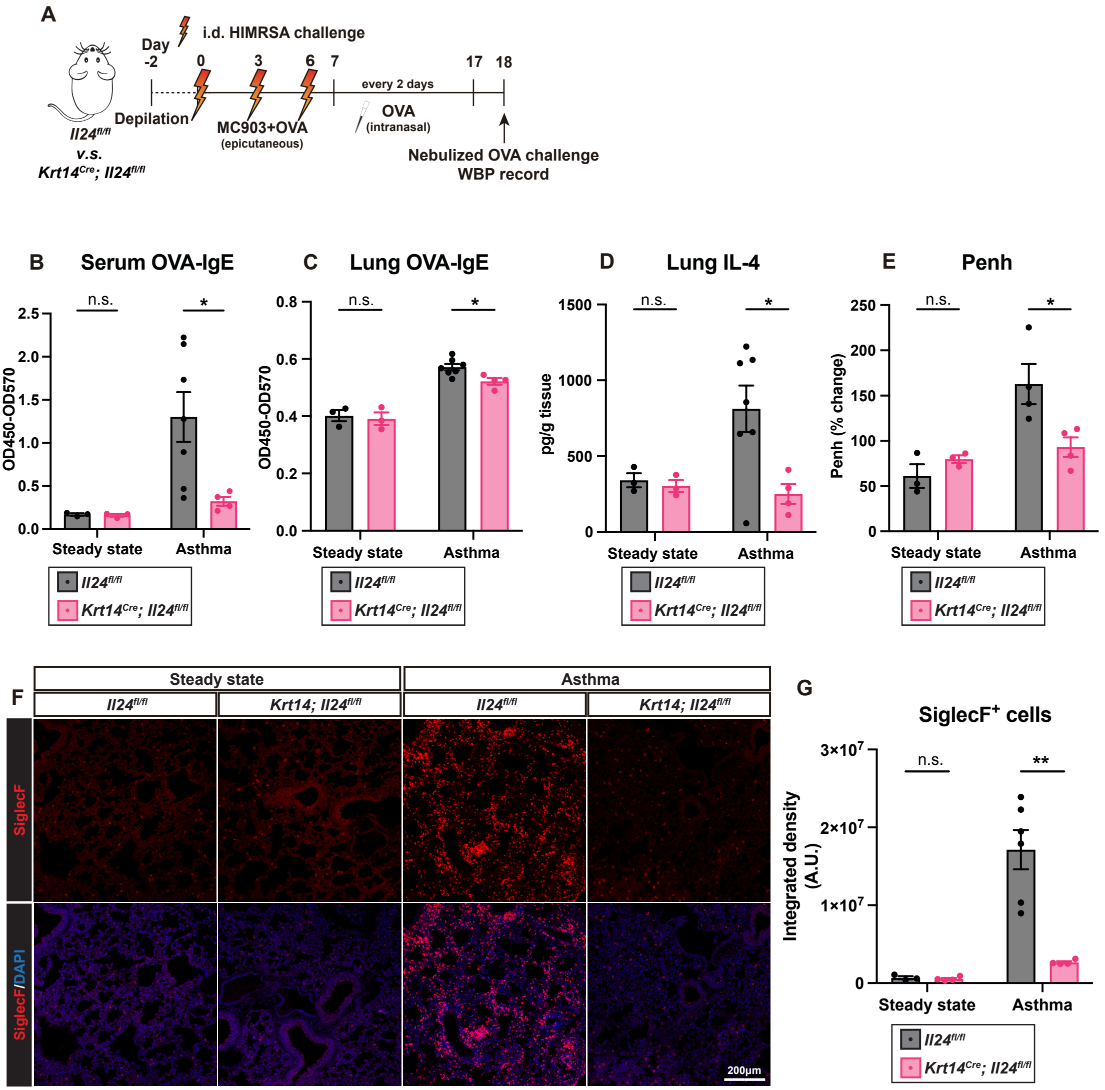
A
Day
-2
i.d. HIMRSA challenge
0
3
6
7
17
18
every 2 days
Depilation
MC903+OVA
(epicutaneous)
OVA
(intranasal)
Nebulized OVA challenge
WBP record
Il24fl/fl
v.s.
Krt14Cre; Il24fl/fl
B
Serum OVA-IgE
C
Lung OVA-IgE
D
Lung IL-4
E
Penh
OD450-OD570
pg/g tissue
Penh (% change)
n.s.
*
Steady state
Asthma
Il24fl/fl
Krt14Cre; Il24fl/fl
F
Steady state
Asthma
Il24fl/fl
Krt14; Il24fl/fl
SiglecF
SiglecF/DAPI
200μm
G
SiglecF+ cells
Integrated density (A.U.)
3×10^7
2×10^7
1×10^7
0
n.s.
**
Steady state
Asthma
Il24fl/fl
Krt14Cre; Il24fl/fl

Fig. S5. Cutaneous administration of recombinant IL-24 exacerbates AD-like inflammation, related to Fig. 3.

A, Schematic of mouse AD-like disease model (MC903+OVA) and experimental pipeline. $Il24^{fl/fl}$ and $Krt14^{Cre}$; $Il24^{fl/fl}$ mice were intradermally (i.d.) treated with PBS or rhIL-24 respectively every day.

B, Ear thickness change between day 0 and day 10 post model induction. Each data point represente the right ear thickness from one individual mouse. n= 3-4 mice. *p < 0.05 by unpaired t test. n.s., not significant.

C, Number of scratching bouts in rhIL-24-treated $Krt14^{Cre}$; $Il24^{fl/fl}$ group and vehicle-treated $Il24^{fl/fl}$ group before i.d. OVA challenge (chronic spontaneous itch; **left**) and following i.d. OVA challenge (acute itch flares; **right**) on day 10 post model induction. n= 4-5 mice, mean ± SEM. n.s., not significant by unpaired t test.

D, Serum IgE (**left**), tissue IgE (**middle**), and tissue IL-4 (**right**) in rhIL-24-treated $Krt14^{Cre}$; $Il24^{fl/fl}$ group and vehicle-treated $Il24^{fl/fl}$ group. n= 3-4 mice, mean ± SEM. n.s., not significant by unpaired t test.

E and F, Representative immunofluorescent images showing the ear of rhIL-24-treated $Krt14^{Cre}$; $Il24^{fl/fl}$ group and vehicle-treated $Il24^{fl/fl}$ group. Anti-keratin 14 antibody was used to label $K14^+$ keratinocytes. The epidermal thickness was assessed and quantified in **F**. n= 3 mice, mean ± SEM. **p < 0.01 by unpaired t test. Dashed lines indicate the border of epidermis.

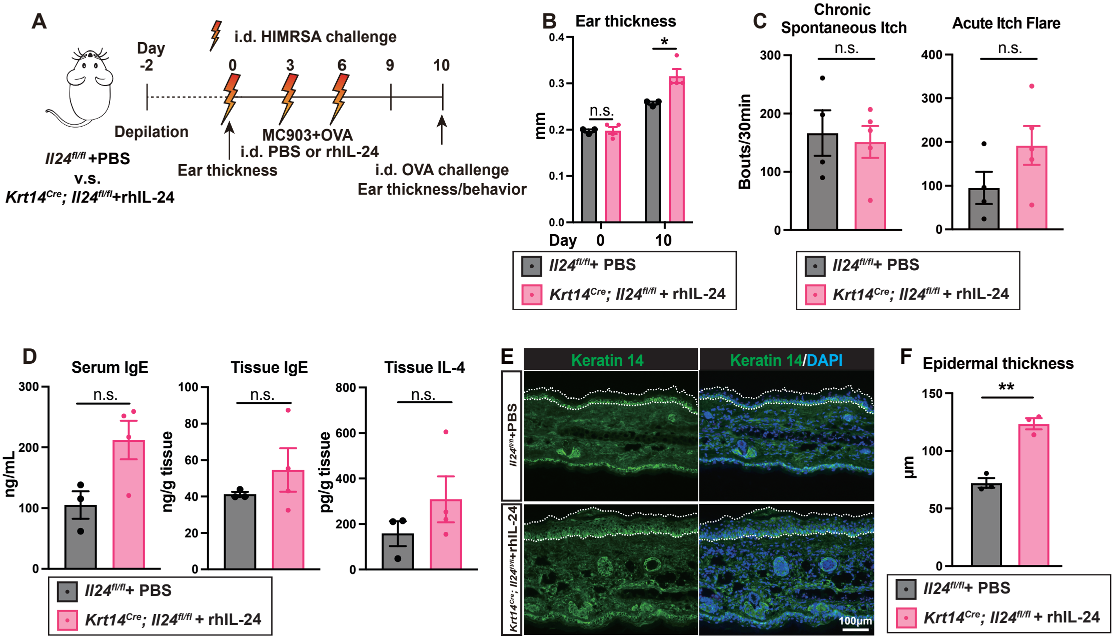
A
Day -2
Depilation
i.d. HIMRSA challenge
0
3
6
9
10
Ear thickness
MC903+OVA
i.d. PBS or rhIL-24
i.d. OVA challenge
Ear thickness/behavior
Il24fl/fl +PBS
v.s.
Krt14Cre; Il24fl/fl+rhIL-24
B
Ear thickness
mm
Day
n.s.
*
C
Chronic Spontaneous Itch
Acute Itch Flare
Bouts/30min
n.s.
Il24fl/fl+ PBS
Krt14Cre; Il24fl/fl + rhIL-24
D
Serum IgE
Tissue IgE
Tissue IL-4
ng/mL
ng/g tissue
pg/g tissue
n.s.
E
Keratin 14
Keratin 14/DAPI
Il24fl/fl+PBS
Krt14Cre; Il24fl/fl+rhIL-24
100μm
F
Epidermal thickness
μm
**

Fig. S6. IL-24 activates JAK-STAT pathway in keratinocytes, related to Fig. 4.
A, Representative immunofluorescent images showing the skin with indicated treatment for 24 hr. Anti-keratin 14 antibody was used to label $K14^+$ keratinocytes.
B, Immunoblot analysis of the skin samples with indicated treatment for 24 hr.
C, Expression of IL-24 receptors of primary keratinocytes over the time course after plating; results were normalized to *Hprt*. n= 3 per time point, mean ± SEM.
D and E, Immunoblot analysis of primary keratinocytes responding to rhIL-24 stimulation for 1 hr. Quantification was shown in **E** indicating the phosphorylation of STAT3 and STAT1. Results were presented by fold normalized to keratin 14. n= 3, mean ± SEM. *p < 0.05, **p < 0.01 by unpaired t test.
F-J, Immunoblot analysis of sorted $SiglecF^+$ (**F**), $SiglecF^-$ (**G**), $CD11c^+$ (**H**), $CD3^+$ (**I**), $CD11b^+$ (**J**) cells responding to rhIL-24 stimulation for 1 hr.
K and L, Immunoblot analysis of cultured BMDC and naïve $CD4^+$ T cells responding to rhIL-24 stimulation for 1 hr.
M, Representative histogram of $GATA3^+$ cells from Th2 cells responding to rhIL-24 stimulation by flow cytometry.

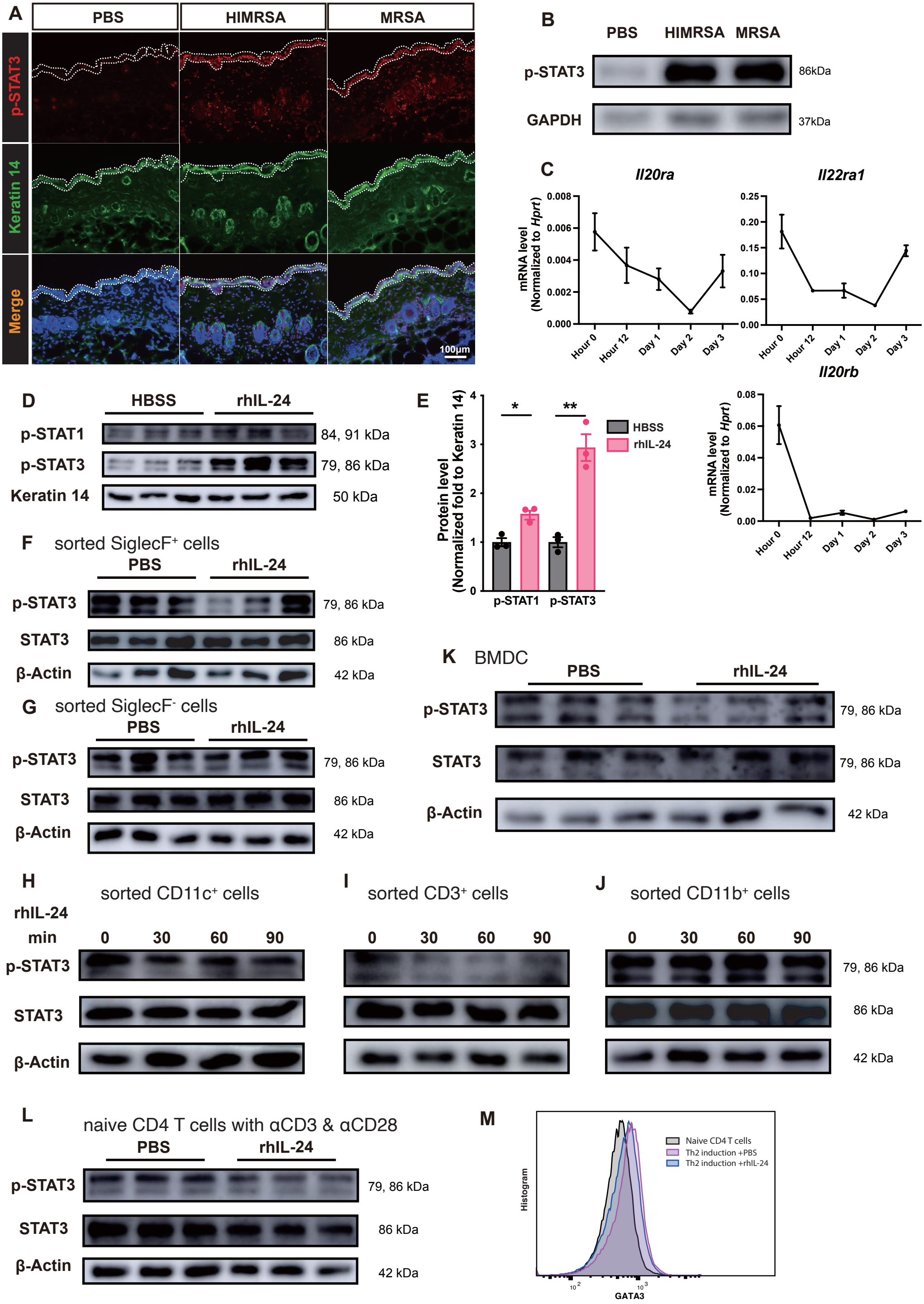

A
PBS
HIMRSA
MRSA
p-STAT3
Keratin 14
Merge
100μm
B
PBS
HIMRSA
MRSA
p-STAT3
86kDa
GAPDH
37kDa
C
Il20ra
Il22ra1
Il20rb
mRNA level
(Normalized to Hprt)
0.008
0.006
0.004
0.002
0.000
0.25
0.20
0.15
0.10
0.05
0.00
0.08
0.06
0.04
0.02
0.00
Hour 0
Hour 12
Day 1
Day 2
Day 3
D
HBSS
rhIL-24
p-STAT1
84, 91 kDa
p-STAT3
79, 86 kDa
Keratin 14
50 kDa
E
Protein level
(Normalized fold to Keratin 14)
4
3
2
1
0
*
**
HBSS
rhIL-24
p-STAT1
p-STAT3
F
sorted SiglecF+ cells
PBS
rhIL-24
p-STAT3
79, 86 kDa
STAT3
86 kDa
β-Actin
42 kDa
G
sorted SiglecF- cells
PBS
rhIL-24
p-STAT3
79, 86 kDa
STAT3
86 kDa
β-Actin
42 kDa
K
BMDC
PBS
rhIL-24
p-STAT3
79, 86 kDa
STAT3
79, 86 kDa
β-Actin
42 kDa
H
sorted CD11c+ cells
rhIL-24
min
0
30
60
90
p-STAT3
STAT3
β-Actin
I
sorted CD3+ cells
0
30
60
90
J
sorted CD11b+ cells
0
30
60
90
79, 86 kDa
86 kDa
42 kDa
L
naive CD4 T cells with αCD3 & αCD28
PBS
rhIL-24
p-STAT3
79, 86 kDa
STAT3
86 kDa
β-Actin
42 kDa
M
Naive CD4 T cells
Th2 induction +PBS
Th2 induction +rhIL-24
Histogram
10^2
10^3
GATA3

Fig. S7. IL-24 exacerbates AD-like inflammation by activating JAK-STAT pathway primarily in K10^{+} keratinocytes, related to Fig. 5.

A-B, Representative immunofluorescent images showing the p-STAT1 signals from *Il24*$^{fl/fl}$ and *Krt14*^{Cre}*; Il24*$^{fl/fl}$ mice with indicated treatment post model induction. Quantification of p-STAT1 and epidermal thickness were shown in **B**. Anti-keratin 14 antibody was used to label K14^{+} keratinocytes. n= 4 mice, mean ± SEM. *p < 0.05, **p < 0.05, ***p < 0.001, ****<0.0001 by unpaired t test. n.s., not significant. Dashed lines indicate the border of epidermis.

C, Dot plot showing the expression of specific top marker genes for keratinocyte-subclusters. KC, keratinocyte.

D and E, Representative immunofluorescent images showing the p-STAT1 signals of the skin from postnatal day 0 (p0) mouse with indicated treatment for 1 hr. Zoomed views were shown in **E**. Anti-keratin 10 antibody was used to label K10^{+} keratinocytes. Dashed lines indicate the border of epidermis and K10^{+} keratinocytes.

F and G, Representative immunofluorescent images showing the p-STAT1 signals of the skin from p0 mouse with indicated treatment for 1 hr. Zoomed views were shown in **G**. Anti-keratin 14 antibody was used to label K14^{+} keratinocytes. Dashed lines indicate the border of epidermis and K14^{+} keratinocytes.

H, Quantification of p-STAT1 signals in epidermis (**upper**) across different types of keratinocytes (**bottom**) after rhIL-24 treatment for 1 hr. n= 10, mean ± SEM. **p<0.01, ****p < 0.0001 by unpaired t test. n.s., not significant. BM, basement membrane.

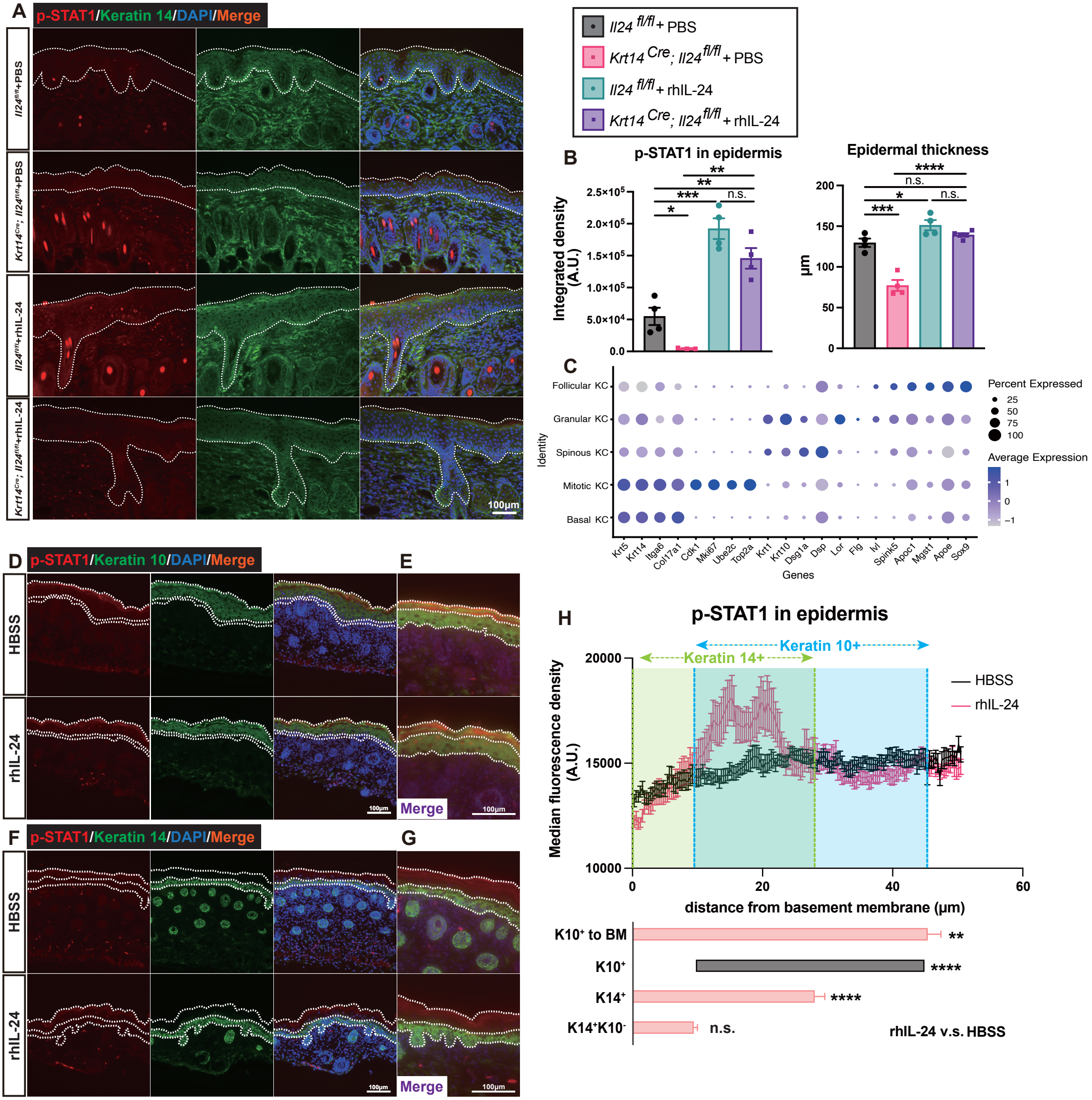

A p-STAT1/Keratin 14/DAPI/Merge
Il24fl/fl+PBS
Krt14Cre; Il24fl/fl+PBS
Il24fl/fl+rhIL-24
Krt14Cre; Il24fl/fl+rhIL-24
100μm
Il24 fl/fl + PBS
Krt14 Cre; Il24 fl/fl + PBS
Il24 fl/fl + rhIL-24
Krt14 Cre; Il24 fl/fl + rhIL-24
B
p-STAT1 in epidermis
Integrated density (A.U.)
Epidermal thickness
μm
n.s.
C
Follicular KC
Granular KC
Spinous KC
Mitotic KC
Basal KC
Identity
Genes
Percent Expressed
Average Expression
Krt5 Krt14 Itga6 Col17a1 Cdk1 Mki67 Ube2c Top2a Krt1 Krt10 Dsg1a Dsp Lor Flg Ivl Spink5 Apoc1 Mgst1 Apoe Sox9
D p-STAT1/Keratin 10/DAPI/Merge
E
HBSS
rhIL-24
Merge
F p-STAT1/Keratin 14/DAPI/Merge
G
H
p-STAT1 in epidermis
Keratin 10+
Keratin 14+
Median fluorescence density (A.U.)
distance from basement membrane (μm)
HBSS
rhIL-24
K10+ to BM
K10+
K14+
K14+K10-
n.s.
rhIL-24 v.s. HBSS

Fig. S8. Keratinocyte specific *Il20rb* depletion abrogates IL-24-STAT3 signaling, related to Fig. 6.

A, Schematic graph showing the strategy for generating *Krt14*^{Cre}*; Il20rb*$^{fl/fl}$ conditional knockout mice.

B and C, Deletion of *Il20rb* exon 2 in keratinocytes in *Krt14*^{Cre}*; Il20rbf*$^{fl/fl}$ mice, determined by DNA-qPCR (**B**) and RT-qPCR (**C**). n=3-7 mice, mean ± SEM. **p< 0.01, ****p < 0.0001 by unpaired t test.

D and E, Immunoblot analysis of *Krt14*^{Cre}*; Il20rbf*$^{fl/fl}$ and littermate control *Il20rbf*$^{fl/fl}$ mice responding to rhIL-24 stimulation for 1 hr. Quantification was shown in **E** indicating the phosphorylation of STAT3. Results were presented as normalized to β-Actin. n= 3, mean ± SEM. *p < 0.05, **p < 0.01 by unpaired t test. n.s., not significant.

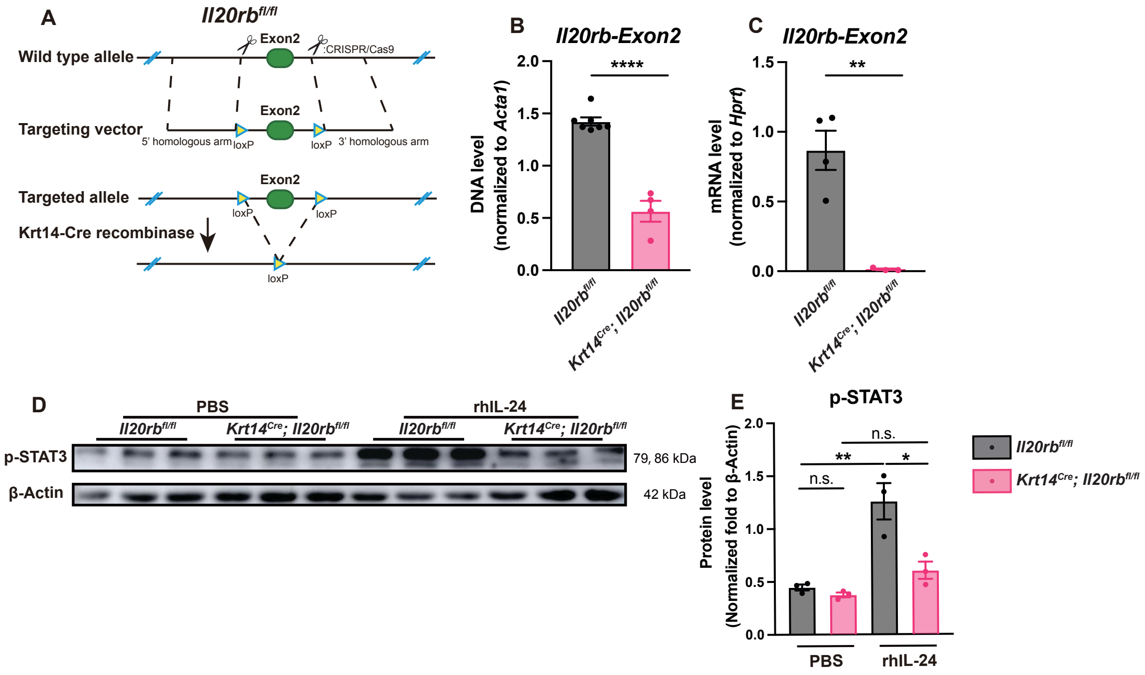

A
Il20rbfl/fl
Wild type allele
Exon2
:CRISPR/Cas9
Targeting vector
Exon2
5' homologous arm
loxP
loxP
3' homologous arm
Targeted allele
Exon2
loxP
loxP
Krt14-Cre recombinase
loxP
B
Il20rb-Exon2
DNA level (normalized to Acta1)
****
Il20rbfl/fl
Krt14Cre; Il20rbfl/fl
C
Il20rb-Exon2
mRNA level (normalized to Hprt)
**
Il20rbfl/fl
Krt14Cre; Il20rbfl/fl
D
PBS
rhIL-24
Il20rbfl/fl
Krt14Cre; Il20rbfl/fl
Il20rbfl/fl
Krt14Cre; Il20rbfl/fl
p-STAT3
79, 86 kDa
β-Actin
42 kDa
E
p-STAT3
Protein level (Normalized fold to β-Actin)
n.s.
**
*
n.s.
PBS
rhIL-24
Il20rbfl/fl
Krt14Cre; Il20rbfl/fl

Fig. S9. IL-24 enhances IL-33 production and drives type 2 immunity, related to Fig. 7.

A, KEGG pathway analysis of shared DEGs in skin samples from rhIL-24-treated mice (8 hr) and vehicle-control by RNA-Seq.

B, Volcano plot displaying genes that were differentially expressed in skin samples from rhIL-24-treated mice (8 hr) and vehicle-control by RNA-Seq. Dotted horizontal lines and dotted vertical lines represent, respectively, significance ($FDR < 0.05$) and fold change ($\log2\ FC > |0.5|$) thresholds.

C, Expression of *Flg, Tslp and Postn* mRNA in the skin of *wild-type* mice with indicated treatment for 12 hr. Relative expression level was normalized to *Hprt*. n= 4 mice, mean ± SEM. *$p < 0.05$, ***$p < 0.001$ by unpaired t test.

D and E, Representative immunofluorescent images showing the IL-33 signals from *Il24*$^{fl/fl}$ and *Krt14*^{Cre}*; Il24*$^{fl/fl}$ mice with indicated treatment after AD-like disease model induction. Quantification of IL-33 were shown in **E**. n= 3-4 mice, mean ± SEM. *$p < 0.05$ by unpaired t test. n.s., not significant. Dashed lines indicate the border of epidermis.

F, *De novo* motif analysis identifying the highly representative STAT3 and STAT1 consensus DNA-recognition motif in wounded tissue and hypoxia-induced keratinocytes reanalyzed from a publicly available dataset. (NCBI BioProject accession no. PRJNA731164)

G and H, Representative immunofluorescent images showing the IL-33 signals from *Il20rb*$^{fl/fl}$ and *Krt14*^{Cre}*; Il20rb*$^{fl/fl}$ mice with indicated treatment after model induction. Quantification of IL-33 were shown in **H**. n= 4-7 mice, mean ± SEM. *$p < 0.05$ by unpaired t test. Dashed lines indicate the border of epidermis.

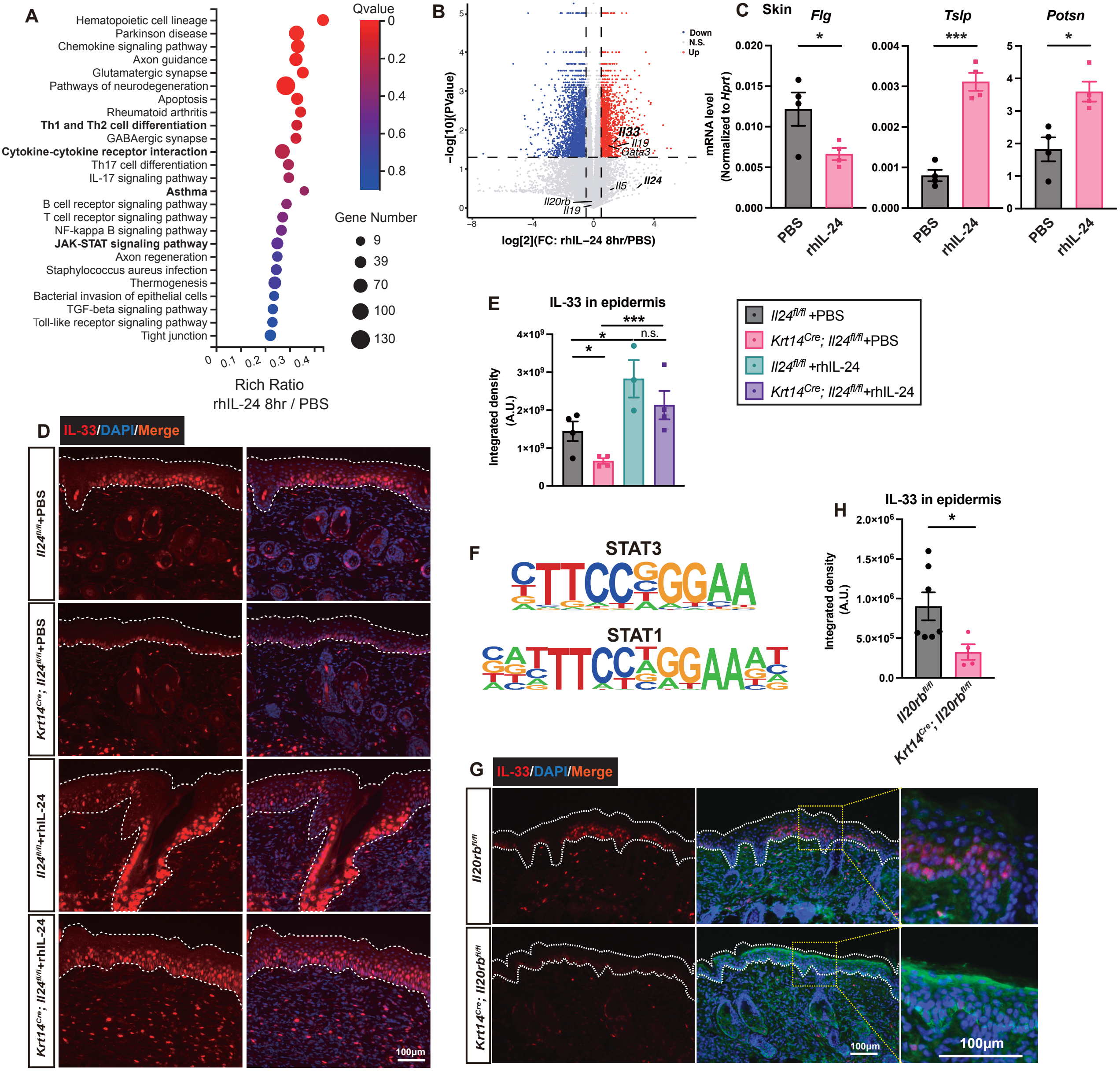
A
Hematopoietic cell lineage
Parkinson disease
Chemokine signaling pathway
Axon guidance
Glutamatergic synapse
Pathways of neurodegeneration
Apoptosis
Rheumatoid arthritis
Th1 and Th2 cell differentiation
GABAergic synapse
Cytokine-cytokine receptor interaction
Th17 cell differentiation
IL-17 signaling pathway
Asthma
B cell receptor signaling pathway
T cell receptor signaling pathway
NF-kappa B signaling pathway
JAK-STAT signaling pathway
Axon regeneration
Staphylococcus aureus infection
Thermogenesis
Bacterial invasion of epithelial cells
TGF-beta signaling pathway
Toll-like receptor signaling pathway
Tight junction
Qvalue
Gene Number
Rich Ratio
rhIL-24 8hr / PBS
B
−log[10](PValue)
log[2](FC: rhIL−24 8hr/PBS)
Down
N.S.
Up
Il33
Il19
Gata3
Il5
Il24
Il20rb
Il19
C Skin
Flg
Tslp
Potsn
mRNA level
(Normalized to Hprt)
PBS
rhIL-24
D IL-33/DAPI/Merge
Il24fl/fl+PBS
Krt14Cre; Il24fl/fl+PBS
Il24fl/fl+rhIL-24
Krt14Cre; Il24fl/fl+rhIL-24
100μm
E IL-33 in epidermis
Integrated density
(A.U.)
n.s.
Il24fl/fl +PBS
Krt14Cre; Il24fl/fl+PBS
Il24fl/fl +rhIL-24
Krt14Cre; Il24fl/fl+rhIL-24
F
STAT3
STAT1
H IL-33 in epidermis
Integrated density
(A.U.)
Il20rbfl/fl
Krt14Cre; Il20rbfl/fl
G IL-33/DAPI/Merge
Il20rbfl/fl
Krt14Cre; Il20rbfl/fl
100μm
100μm

Fig. S10. Sensory neurons do not express the receptors of IL-24.

A, Expression of IL-24 receptors and itch-related transcripts by mouse DRG neuron populations based on published single-cell RNA-seq datasets(Zeisel et al., 2018).

Mrgpra3, MAS-related GPR member A3; *Hrh1*, histamine receptor H1; *S1pr3*, sphingosine-1-phosphate receptor 3.

B, Expression of *Il20ra*, *Il22ra1*, *Il20rb* mRNA in the DRG of *wild-type* mice.

C, scRNA-Seq data from Tavares-Ferreira et al.(Tavares-Ferreira et al., 2022) demonstrates that the expression of *Il20RA*, *Il22RA1*, *Il20RB* were barely detectable in pruriceptors which highly express *GFRA2*, *IL31RA*, and *NPPB*. Data are presented as estimated counts from Seurat analysis. *GFRA2*, GDNF family receptor alpha 2; *IL31RA*, interleukin-31 receptor alpha; *NPPB*, natriuretic peptide B.

A Mouse DRG neurons

	Peptidergic								Neurofilament			Non-peptidergic					
	TrpM8	TrpM8	TrpM8	PEP1.2	PEP1.3	PEP1.1	PEP1.4	PEP2	NF2/3	NF4/5	NF1	TH	NP1.1	NP1.2	NP2.1	NP2.2	NP3
Il20ra	0.00	0.00	0.00	0.00	0.00	0.00	0.00	0.00	0.00	0.00	0.00	0.00	0.00	0.00	0.00	0.00	0.00
Il20rb	0.00	0.00	0.00	0.04	0.02	0.01	0.02	0.01	0.00	0.00	0.00	0.02	0.00	0.00	0.00	0.02	0.00
Il22ra1	0.00	0.00	0.00	0.00	0.00	0.00	0.00	0.00	0.00	0.00	0.00	0.00	0.00	0.00	0.00	0.00	0.00
Mrgpra3	0.00	0.00	0.00	0.00	0.00	0.00	0.01	0.00	0.00	0.10	0.00	0.00	0.00	0.01	8.40	3.30	0.06
Hrh1	0.00	0.00	0.00	0.00	0.12	0.00	0.02	0.01	0.10	0.00	0.03	0.01	0.00	0.01	0.25	0.63	0.47
S1pr3	0.07	0.05	0.11	0.57	3.54	1.29	2.05	2.89	2.32	0.26	0.97	0.07	0.06	0.08	0.00	0.10	0.51

B

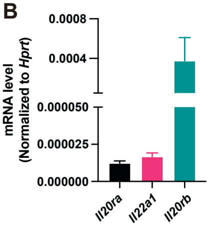

C Human DRG neurons

	C-LTMRs	Nociceptors							Mechanoreceptors			Proprioceptors
		Pruritogen receptor	Silent nociceptors	TRPA1+	PENK+	Aδ HTMRs	Cold nociceptors	Aß nociceptors	Aδ LTMRs	Aß RA LTMRs	Aß SA LTMRs	
GFRA2	1.58	2.71	0.12	0.11	0.11	0.11	0.10	0.22	0.25	0.27	0.10	0.08
IL31RA	1.23	2.13	4.92	0.36	0.10	0.09	0.14	0.13	0.14	0.10	0.09	0.10
NPPB	0.27	0.57	0.60	0.04	0.01	0.02	0.01	0.05	0.01	0.03	0.01	0.02
IL20RA	0.02	0.01	0.00	0.01	0.01	0.02	0.06	0.00	0.00	0.01	0.00	0.00
IL20RB	0.07	0.04	0.03	0.06	0.05	0.04	0.05	0.06	0.05	0.04	0.06	0.05
IL22RA1	0.00	0.00	0.00	0.00	0.00	0.00	0.00	0.00	0.00	0.00	0.00	0.00

Video S1. MC903 and OVA induced AD-like skin inflammation, exhibiting skin redness and itching behavior.

Video S2 and S3. Representative immunofluorescent images showing the p-STAT3 signals(**red**) of the skin from p0 mouse with rhIL-24 (**S3**) or HBSS (**S2**) control treatment for 1 hr. Anti-keratin 14 antibody was used to label $K14^+$ keratinocytes (**green**), DAPI was used to label nuclei (**blue**).

Table S1. Top 10 feature genes of each cell type

Cell type	Top 10 feature genes
Keratinocytes	*Cst6/Lgals7/Krt14/Krt17/Krtdap/Ly6d/Perp/Defb6/Sfn/Krt5*
Fibroblasts	*Saa3/Dcn/Col1a2/Col1a1/Col3a1/Tnfaip6/Tnc/Sparc/Aebp1/Mmp3*
Endothelial cells	*Ccl21a/Ccl21a/Lyve1/Lcn2/Mmrn1/Fgl2/Lrg1/Gdf15/Cldn5/Nts*
Macrophages	*S100a9/S100a8/Retnlg/Ccl4/Ccl3/Cxcl2/Wfdc17/Ccl8/Il1b/G0s2*
Dendritic cells	*H2-Eb1/Cd74/H2-Ab1/Cd207/H2-Aa/Pgf/Mfge8/Cd86/Malt1/Il1r2*
ILC, NK, T cells	*Ctla2a/Rgs1/Zeb1/Tox/Itk/Grap2/Trdc/Dennd4a/Prkch/Abcb1b*

List of qPCR primers

Primers for qPCR

mIl24-F CTGGACTGTGAAGAACACTGTGCA
mIl24-R AGCTCTCAGCACCCGAGACA
mIl19-F TGGAGAACCTCAGGAGCATT
mIl19-R GAATGTCAGCAGGTTGTTGG
mIl20-F CTCCTTTAACTGGGCTCAAGAC
mIl20-R CACCTATCCAAAGACACACTATCC
mIl1b-F AGTTGACGGACCCCAAAAG
mIl1b-R AGCTGGATGCTCTCATCAGG
mIl33-F GGTGAACATGAGTCCCATCA
mIl33-R CGTCACCCCTTTGAAGCTC
mTslp-F TCATGACCTGACTGGAGATTTG
mTslp-R AGCCAGGGATAGGATTGAGA
mCxcl-F GTGTCTAGTTGGTAGGGCATAAT
mCxcl-R CAGTCCTTTGAACGTCTCTGT
mS100a8-F GAACTGGAGAAGGCCTTGAG
mS100a8-R CTTGTAGAGGGCATGGTGATT
mDefb4-F GCCTTGGAAAGGAAGAACAAATAG
mDefb4-R CCCTCACCCTGCATATTCTAAG
mSerpinb3a-F GAAGGCTGACTTCTCTGGTATG
mSerpinb3a-R CTGTTCCCTCTTCATTCACCTC
mPostn-F CTCGGACTGTGATGCCTTAAT
mPostn-R TGGATCCACACCTTGCATTTA
mKrt14-F GGTGGTCGATTTGATGGATTTG
mKrt14-R CAGGAGCCCATCACCAATAC
mIl24-exon2-F AGAGTTCCGATTTGGGTCTTG
mIl24-exon2-R GCTTGTGATGTCATCCTGAGT
mIl20ra-F GCCAAAGTGAAGGCCATTTG

mIl20ra-R GGTGGGCTGACTTGAGTTT
mIl22ra1-F TCCAGTCCAGCAACTTTGAG
mIl22ra1-R GCCACTTTCTCTCTCCGTATTT
mIl20rb-F GACCATCCAAGAGTGACAGAAA
mIl20rb-R GTCCGAGGTATGAAGCCATTT
mIl20rb-exon2-F GAACCTCTCTGTACAGTCAACC
mIl20rb-exon2-R CCCTGATACTCCACACAATAGAG
mFlg-F AATCCCACTCCAAACCATCTC
mFlg-R TTCCTCCTGATCCCGATCTT
mMki67-F GAGGAGAAACGCCAACCAAGAG
mMki67-R TTTGTCCTCGGTGGCGTTATCC
mHprt-F TGAAGAGCTACTGTAATGATCAGTCAAC
mHprt-R AGCAAGCTTGCAACCTTAACCA
mActa1-F CGCTGTGGCGTCCTATAAA
mActa1-R AACGAAGGAGCTGCAAAGA

List of HCR probes and hairpins

anti-sense probes for *Il24* mRNA

anti-sense probe-1 GATCCTCGTACTCATTCCTTggaaatgatttcaaaagtgaaagac
anti-sense probe-2 ctgggtgggaagctccttctcagctTTCACCTTTACATCACCTCG
anti-sense probe-3 GATCCTCGTACTCATTCCTTcagcattcattggtagaggatcttc
anti-sense probe-4 gtgcacaaaaagttgggcaggctcaTTCACCTTTACATCACCTCG
anti-sense probe-5 GATCCTCGTACTCATTCCTTgctgtgtgaaggtggctggttcttc
anti-sense probe-6 tagggtcctaaagtgaagccggaggTTCACCTTTACATCACCTCG
anti-sense probe-7 GATCCTCGTACTCATTCCTTtgttgaagaaagggccagtgctcct
anti-sense probe-8 gagaatctgtagtccccaactcatcTTCACCTTTACATCACCTCG
anti-sense probe-9 GATCCTCGTACTCATTCCTTaagaagaaggattaggctcaggcag
anti-sense probe-10 accctcaagccctggcacttggttcTTCACCTTTACATCACCTCG
anti-sense probe-11 GATCCTCGTACTCATTCCTTttggcaagacccaaatcggaactct
anti-sense probe-12 cagttctgggagaaccacccctgtcTTCACCTTTACATCACCTCG
anti-sense probe-13 GATCCTCGTACTCATTCCTTgttcttcacagtccagaaggcctcc
anti-sense probe-14 tgtgatgtcatcctgagtttgcacaTTCACCTTTACATCACCTCG
anti-sense probe-15 GATCCTCGTACTCATTCCTTaacctgcggcttcaacagccggatg
anti-sense probe-16 gctctcagcacccgagacattccgcTTCACCTTTACATCACCTCG
anti-sense probe-17 GATCCTCGTACTCATTCCTTcttcagcaggctgtgggcaaggtaa
anti-sense probe-18 gttcttgaaaacagtgttcaagtagTTCACCTTTACATCACCTCG
anti-sense probe-19 GATCCTCGTACTCATTCCTTcttgaatttggctattttgctgtgg
anti-sense probe-20 ggccagagtggagaatgacctcaagTTCACCTTTACATCACCTCG
anti-sense probe-21 GATCCTCGTACTCATTCCTTttgtgacatgatgactatgaagttg
anti-sense probe-22 catggaattgtccttactgggctgtTTCACCTTTACATCACCTCG
anti-sense probe-23 GATCCTCGTACTCATTCCTTctggtgtgcactctcactaatggga
anti-sense probe-24 gaatgctctgcggaacagcaaaaacTTCACCTTTACATCACCTCG
anti-sense probe-25 GATCCTCGTACTCATTCCTTcaaagcgacttctgtatccaactgt
anti-sense probe-26 aatgtccacttccccaaaggctttcTTCACCTTTACATCACCTCG
anti-sense probe-27 GATCCTCGTACTCATTCCTTgtagaatttctgcatccaggtcagg
anti-sense probe-28 aagttatccaatcagcagtcagagaTTCACCTTTACATCACCTCG

anti-sense probe-29 GATCCTCGTACTCATTCCTTttgaaatggcatggagagcaaagga
anti-sense probe-30 gaggacagcagggatgtacacaatgTTCACCTTTACATCACCTCG
anti-sense probe-31 GATCCTCGTACTCATTCCTTccatggccaagggtctgaagtgcc
anti-sense probe-32 gaggaaaagcctgagccaacaacggTTCACCTTTACATCACCTCG
anti-sense probe-33 GATCCTCGTACTCATTCCTTgtcatttggactgaagagtgaggt
anti-sense probe-34 ggagcatccaaaggtgccatctatgTTCACCTTTACATCACCTCG
anti-sense probe-35 GATCCTCGTACTCATTCCTTtatgcaaatctactttgtgggtcag
anti-sense probe-36 acaataatttaatagggctgtaataTTCACCTTTACATCACCTCG
anti-sense probe-37 GATCCTCGTACTCATTCCTTaaataaatacggtttccagggaagg
anti-sense probe-38 atgctttcatggaacttctggtctcTTCACCTTTACATCACCTCG
anti-sense probe-39 GATCCTCGTACTCATTCCTTggaggaggcatggggcactaaattc
anti-sense probe-40 catgctgagccagatcacaggaagtTTCACCTTTACATCACCTCG

sense probes for *Il24* mRNA

sense probe-1 GATCCTCGTACTCATTCCTTagctgagaaggagcttcccacccag
sense probe-2 gtctttcacttttgaaatcatttccTTCACCTTTACATCACCTCG
sense probe-3 GATCCTCGTACTCATTCCTTtgagcctgcccaactttttgtgcac
sense probe-4 gaagatcctctaccaatgaatgctgTTCACCTTTACATCACCTCG
sense probe-5 GATCCTCGTACTCATTCCTTcctccggcttcactttaggacccta
sense probe-6 gaagaaccagccaccttcacacagcTTCACCTTTACATCACCTCG
sense probe-7 GATCCTCGTACTCATTCCTTgatgagttggggactacagattctc
sense probe-8 aggagcactggccctttcttcaacaTTCACCTTTACATCACCTCG
sense probe-9 GATCCTCGTACTCATTCCTTgaaccaagtgccagggcttgagggt
sense probe-10 ctgcctgagcctaatccttcttcttTTCACCTTTACATCACCTCG
sense probe-11 GATCCTCGTACTCATTCCTTgacaggggtggttctcccagaactg
sense probe-12 agagttccgatttgggtcttgccaaTTCACCTTTACATCACCTCG
sense probe-13 GATCCTCGTACTCATTCCTTtgtgcaaactcaggatgacatcaca
sense probe-14 ggaggccttctggactgtgaagaacTTCACCTTTACATCACCTCG
sense probe-15 GATCCTCGTACTCATTCCTTgcggaatgtctcgggtgctgagagc
sense probe-16 catccggctgttgaagccgcaggttTTCACCTTTACATCACCTCG
sense probe-17 GATCCTCGTACTCATTCCTTctacttgaacactgttttcaagaac
sense probe-18 ttaccttgcccacagcctgctgaagTTCACCTTTACATCACCTCG
sense probe-19 GATCCTCGTACTCATTCCTTcttgaggtcattctccactctggcc
sense probe-20 ccacagcaaaatagccaaattcaagTTCACCTTTACATCACCTCG
sense probe-21 GATCCTCGTACTCATTCCTTacagcccagtaaggacaattccatg
sense probe-22 caacttcatagtcatcatgtcacaaTTCACCTTTACATCACCTCG
sense probe-23 GATCCTCGTACTCATTCCTTgtttttgctgttccgcagagcattc
sense probe-24 tcccattagtgagagtgcacaccagTTCACCTTTACATCACCTCG
sense probe-25 GATCCTCGTACTCATTCCTTgaaagcctttggggaagtggacatt
sense probe-26 acagttggatacagaagtcgctttgTTCACCTTTACATCACCTCG
sense probe-27 GATCCTCGTACTCATTCCTTtctctgactgctgattggataactt
sense probe-28 cctgacctggatgcagaaattctacTTCACCTTTACATCACCTCG
sense probe-29 GATCCTCGTACTCATTCCTTcattgtgtacatccctgctgtcctc
sense probe-30 tcctttgctctccatgccatttcaaTTCACCTTTACATCACCTCG
sense probe-31 GATCCTCGTACTCATTCCTTccgttgttggctcaggcttttcctc
sense probe-32 ggcacttcagacccttggccatggaTTCACCTTTACATCACCTCG

sense probe-33 GATCCTCGTACTCATTCCTTcatagatggcacctttggatgctcc
sense probe-34 acctcactcttcagtccaaatgacaTTCACCTTTACATCACCTCG
sense probe-35 GATCCTCGTACTCATTCCTTtattacagccctattaaattattgt
sense probe-36 ctgacccacaaagtagatttgcataTTCACCTTTACATCACCTCG
sense probe-37 GATCCTCGTACTCATTCCTTgagaccagaagttccatgaaagcat
sense probe-38 ccttccctggaaaccgtatttatttTTCACCTTTACATCACCTCG
sense probe-39 GATCCTCGTACTCATTCCTTacttcctgtgatctggctcagcatg
sense probe-40 gaatttagtgccccatgcctcctccTTCACCTTTACATCACCTCG

Hairpin sequences for the probes

H1-(Alexa 488)
CGAGGTGATGTAAAGGTGGGAATGAGTACGAGGATCGACTTGACACTAGATCCTCG
TACTCATTCCCACCTT
H1-(Alexa 488)
GATCCTCGTACTCATTCCCACCTTTACATCACCTCGAAGGTGGGAATGAGTACGAGG
ATCTAGTGTCAAGTC

List of ChIP-qPCR primers

Region1-F TTGGTAGGACCACTTGGGTAG
Region1-R TGATTCCTGGAACAATCTTGTGG
Region2-F GAAGATGCCTCCTTGGTATCAG
Region2-R AGATCACACCAAAGAGATTCCC
Region3-F TAAGCTTCAGAAGGCAGGATTT
Region3-R CAGTGATAGGCTTGAGTCTGTG
Region4-F CGCTCCATTTGCATAACTTTACTC
Region4-R GCTGGTCTTGAATGCTGTACT
Region5-F GCGACTGCTTTCCGCTTA
Region5-R TGATAGCTGATCATGGCTTAGTAG

Tavares-Ferreira, D., Shiers, S., Ray, P.R., Wangzhou, A., Jeevakumar, V., Sankaranarayanan, I., Cervantes, A.M., Reese, J.C., Chamessian, A., Copits, B.A., et al. (2022). Spatial transcriptomics of dorsal root ganglia identifies molecular signatures of human nociceptors. Science Translational Medicine *14*, eabj8186. doi:10.1126/scitranslmed.abj8186.

Zeisel, A., Hochgerner, H., Lonnerberg, P., Johnsson, A., Memic, F., van der Zwan, J., Haring, M., Braun, E., Borm, L.E., La Manno, G., et al. (2018). Molecular Architecture of the Mouse Nervous System. Cell *174*, 999-1014 e1022. 10.1016/j.cell.2018.06.021.
